# C-terminal S-acylation governs membrane distribution, interaction dynamics and function of a plant Rho GTPase

**DOI:** 10.1371/journal.pone.0348444

**Published:** 2026-04-30

**Authors:** Amir Akerman, Orit Gutman, Keren E. Shapira, Efraim Lewinsohn, Yoav I. Henis, Shaul Yalovsky

**Affiliations:** 1 School of Plant Sciences and Food Security, Faculty of Life Sciences, Tel Aviv University, Tel Aviv, Israel; 2 Department of Neurobiology, Faculty of Life Sciences, Tel Aviv University, Tel Aviv, Israel; 3 Department of Field and Vegetable Crops, Agricultural Research Organization, Neve Ya’ar Research Center, Ramat Yishay, Israel; Amity University Mumbai, INDIA

## Abstract

Rho of Plants (ROPs) are plant-specific Rho GTPases that regulate diverse cellular processes. Based on their C-terminal motifs, ROPs are classified as type I or type II. Type-II ROPs lack the canonical CaaL prenylation motif and can be palmitoylated *in vitro*, yet *in vivo* evidence for their S-acylation and functional significance has been limited. Here, we investigated the membrane association, lipid modifications and function of the *Arabidopsis* type-II ROP, ROP10. Confocal microscopy, biochemical fractionation and fluorescence recovery after photobleaching (FRAP) analyses showed that wild-type (WT) ROP10 associates with the plasma membrane in an activation- and Cys199/205-dependent manner, with Cys160 further stabilizes membrane association. Unlike type-I ROPs, the constitutively active (CA) rop10CA mutant displayed reduced membrane affinity. Gas chromatography-mass spectrometry confirmed S-acylation of ROP10 by palmitic and stearic acids. Overexpression of WT ROP10 disrupted cell polarity, an effect abolished in the rop10^C160S^ mutant, establishing Cys160 as essential for polarity control. These results provide *in vivo* evidence for S-acylation of type-II ROPs and indicate divergence from type-I ROPs through partitioning into distinct plasma membrane subdomains.

## Introduction

Plants possess a distinct family of Rho GTPases known as Rho of Plants (ROP) or RACs [1–3]. ROPs have been implicated in a wide range of functions, including the regulation of the actin and microtubule cytoskeleton, vesicle trafficking, abscisic acid and auxin signaling, reactive oxygen species (ROS) formation, response to microbial and fungal pathogens, and nitrogen signaling [4–12].

Based on their amino acid sequences, ROPs have been categorized into two subgroups, designated type-I and type-II [1,3]. Type-I ROPs terminate with a canonical CaaL box motif and are predominantly geranylgeranylated by protein geranylgeranyl transferase I (PGGT-I) (Fig. S1, in S1 File Supporting Information and [13,14]). In contrast, type-II ROPs terminate with a motif known as the GC-CG box. This motif comprises two cysteine residues separated by 5–6 predominantly aliphatic amino acids and adjacent glycine residues positioned either upstream or downstream of the cysteine. It has been demonstrated that type-II ROPs are not prenylated, and their attachment to the plasma membrane necessitates an intact GC-CG box and a proximal polybasic domain. Furthermore, type-II ROPs can be palmitoylated *in vitro* and their attachment to the plasma membrane was reduced by treatment with the protein S-acylation inhibitor 2-bromopalmitate [15,16]. Additionally, type-II ROPs were identified in proteomics analysis of plant S-acylated proteins [17]. However, direct *in vivo* evidence of S-acylation of type-II ROPs and the identity of the acyl moieties with which they are modified have not been presented. Furthermore, previous analysis of type-II ROPs was carried out by transient expression in a heterologous system of *Nicotiana benthamiana* leaf epidermal cells and not in *Arabidopsis*.

The model plant *Arabidopsis thaliana* (*Arabidopsis*) possesses 11 ROPs, of which ROP1 to ROP8 are type-I ROPs and ROP9 to ROP11 are type-II ROPs (S1 Fig, In S1 File Supporting Information and [3]). Studies on the *Arabidopsis* AtROP6 (ROP6), a type-I ROP, have demonstrated that its activated GTP-bound form partitions into a triton X-100-insoluble membrane (TIM) fraction. Further analysis revealed that this partitioning is associated with transient S-acylation of G-domain Cys residues C21 and primarily C158. GTPase assays have confirmed that rop6^C21S^ rop6^C158S^ and rop6^C21S C158S^ point mutants do not exhibit compromised GTPase activity [14,18,19].

We have formerly evaluated the interaction dynamics of ROP6, its constitutively active rop6CA mutant, and the rop6^C21S C158S^ mutant with the membrane by Fluorescence Recovery After Photobleaching (FRAP) beam size analysis [18] (for method, see [20]). These studies have indicated that fluorescence recovery of rop6CA occurred solely through lateral diffusion, while the recovery of wild-type (WT) ROP6 was predominantly characterized by lateral diffusion and a minor contribution of exchange between membrane-bound and cytoplasmic ROP6 molecules. However, the recovery of the rop6^C21S C158S^ mutant was primarily by exchange. Gain-of-function mutant analysis revealed that the rop6CA^C21S C158S^ mutant is not functional, suggesting that the activation-dependent transient S-acylation is essential for ROP6 function [18]. Notably, C21 and C158 are conserved between different ROPs and even in non-plant Rho GTPases from evolutionarily distant organisms (Fig. S2, in S1 File Supporting Information), implying a potential conservation of their function.

When coexpressed with a ROP Guanyl nucleotide Exchange Factor (ROPGEF) and a ROP GTPase Activating Protein (ROPGAP), ROPs form self-organizing membrane domains [21,22]. Coexpression of the microtubule-binding ROP effector Interactor of Constitutively Active ROP 1 (ICR1) revealed that type-I and type-II ROPs possess distinct functionalities within these domains. All three type-II ROPs (ROP9, ROP10, and ROP11) induced the microtubules rearrangement associated with ICR1 around the domains. However, ICR1 microtubules were not recruited to the type-I ROP2 and ROP4 domains. Nevertheless, the ICR1 that was recruited to ROP6 domains was not associated with microtubules [22]. These findings suggested that type-I and type-II ROPs may have distinct functions.

The membrane partitioning and interaction dynamics of type-II ROPs with the plasma membrane, and the functional consequences of these interactions, remain insufficiently characterized. Therefore, the identity of the acyl moieties in vivo, the dynamics of membrane interaction, and the functional consequences for a type-II ROP remained unknown. In this study, we investigated the plasma membrane association and dynamics of ROP10 following stable expression in Arabidopsis. Lipid modifications of purified recombinant ROP10 were analyzed using gas chromatography-mass spectrometry (GC-MS) [19]. We then examined the membrane interaction dynamics of ROP10 (WT and selected point mutants) using FRAP beam-size analysis and subcellular localization assays. These analyses revealed that wild-type ROP10 exhibits relatively strong membrane interactions, which are weakened in the constitutively active, GTP-bound form (rop10CA). Notably, the reduction in membrane interaction was further enhanced in rop10CA^C160S^ and rop10CA^C23S^, indicating that these cysteine residues contribute to membrane association in the activated state. Functional analyses based on gain-of-function assays showed that altered membrane interactions correlate with reduced ROP10 activity. Together, our results indicate that ROP10 is S-acylated by palmitic and stearic acids, and suggest that its membrane interaction properties differ from those of the type-I ROP6. Furthermore, C160 (the residue orthologous to C158 in ROP6) is critical for ROP10 function.

## Materials and Methods

**Biological materials.** All plasmids, bacterial strains, oligonucleotide primers and transgenic plants used in this work are listed in Supplemental Tables S1, S2, S3 and S4, respectively, in Supporting Information S1 File. Requests for materials should be addressed to the corresponding author.

**Molecular cloning.** GFP-ROP10, GFP-rop10CA, GFP-rop10^Δ183-197^, GFProp10^Δ200-204^, and GFP-rop10^C199S C205S^ [15,16] were used to generate His_6_-GFP-ROP10, His_6_-GFP-rop10CA, His_6_-GFP-rop10^Δ183-197^, His_6_-GFProp10^Δ200-204^, and His_6_-GFP-rop10^C199S C205S^. The ROP10 cDNAs were digested with SacI and XbaI and subcloned into pRTL-His_6_-GFP. Subsequently, the His_6_-GFP-ROP10 cassettes were isolated by digestion with HindIII and subcloned into pCAMBIA2300 and pCAMBIA3300 plant binary vectors. The C23S and C160S ROP10 and rop10CA mutants were generated by site-directed mutagenesis of respective plasmids as previously described [15]. All constructs were verified by sequencing.

**Plant genomic DNA isolation.** Typically, 100–200 mg of leaf tissue were ground using a mortar and pestle. Subsequently, genomic DNA is extracted using the GenElut Plant Genomic DNA Miniprep Kit, following the manufacturer’s protocol (Sigma-Aldrich, Stenheim, Germany).

**RNA isolation.** *Arabidopsis* seedlings were batch-frozen using liquid nitrogen, subsequently ground into a fine powder. Total RNA was subsequently extracted from the ground material using the SV total RNA isolation commercial kit (Promega, Madison, WI, USA), as per the manufacturer’s protocol.

**cDNA synthesis.** Isolation of RNA RT-PCR and cDNA synthesis were carried out as previously described [15].

**Bacterial strains.** DH5α(F’)-*F’* was used for heat shock transformation and molecular manipulation. *Agrobacterium tumefaciens* strain GV3101 pMP90 was used for expression of recombinant genes in *Arabidopsis*.

**SDS-PAGE and immunoblot analysis.** Proteins were resolved on 10% SDS-PAGE [23]. Immunoblots were decorated with mouse αGFP monoclonal antibodies (Covance; cat #MMS-118P) at dilution 1:3,000 as previously described [14,18].

**Preparation of *Arabidopsis* protein extracts for GC-MS analysis.** To prepare protein extracts containing the His_6_-GFP-ROP10 (ROP10) or untransformed plants, 15 g of rosette leaves from 2-week-old transgenic plants was harvested and batch frozen in liquid nitrogen. Proteins were extracted from the frozen leaves by grinding the tissue with a pestle and mortar in 3X volumes (45 mL) of plant protein extraction buffer No.1 (50 mM HEPES-KOH pH 7.5, 2 mM MgCl_2_, 300 mM NaCl, 10% glycerol, 2 mM ß-Mercaptoethanol, plant protease inhibitor mixture (Sigma, St. Louis, MO) and 1mM PMSF). To precipitate insoluble material, extracts were centrifuged at 100,000 *x g* for 60 minutes at 4°C. The insoluble pellet of His_6_-GFP-ROP10 (a membrane protein) was incubated on ice for 30 minutes in the same volume (45 mL) of plant extraction buffer No. 2 containing: 1% Triton X-100 and 0.1% SDS. Solubilized extracts were centrifuged again at 80,000 *x g* for 60 minutes at 4°C. Supernatants containing Triton X-100-insoluble and SDS-soluble fractions were collected for further analysis.

**Purification of His**_**6**_**-GFP-ROP10.** All protein purifications were conducted using the AKTA-prime protein purification system (GE Healthcare, UK). Prior to final purification, the His_6_-GFP-ROP10 recombinant protein underwent a 24-hour dialysis at 4°C, stirring against 5 liters of dialysis buffer No. 1, which contained: 50 mM Tris-HCl (pH 8.5), 2 mM MgCl_2_, 25 mM NaCl, 10% glycerol, 2 mM β-mercaptoethanol, 0.5% Triton X-100, and 1 mM PMSF. The dialyzed fractions were collected, and the His_6_-GFP-ROP10 protein was purified over a Ni-NTA column equipped with a His-TRAP FF column (GE Healthcare, UK) of 25 mL bed volume. After multiple repeated experiments, the purified protein quantity was insufficient for lipid analysis by GC-MS. To address this issue, the proteins underwent enrichment by anion exchange chromatography using a 25 mL HiTrap Q FF column (GE Healthcare, UK). The column was pre-washed with 250 mL of wash buffer prior to loading the protein sample. After loading, the column was washed with 250 mL of wash buffer and 10 mL fractions were eluted using a linear elution buffer gradient (25–800 mM NaCl). Subsequently, a second dialysis was performed on the fraction containing the enriched protein to reduce its salt concentration prior to the S-acylation analysis. This dialysis was conducted for 24 hours at 4°C, stirring, against 1 liter of dialysis buffer No. 2, which contained: 20 mM HEPES-KOH (pH 7.5), 1 mM MgCl_2_, 25 mM NaCl, 5% glycerol, 2 mM β-mercaptoethanol, 0.1% Triton X-100, and 1 mM PMSF. Protein concentrations were determined using the BCA Protein Assay kit (Pierce, Rockford, IL 61105 USA) according to the manufacturer’s protocol. The same enrichment protocol was applied to untransformed plant protein extracts, which served as mock controls in the GC-MS analysis.

**Partitioning of His6-GFP-ROP10 and its mutants.** To analyze the partitioning of His_6_-GFP-ROP10 (ROP10) and all its respective mutants, 300 mg of cotyledons from 10-day-old transgenic plants were harvested and batch-frozen in liquid nitrogen. Subsequently, proteins were extracted from the frozen leaves by grinding the tissue with a pestle and mortar in three times the volume of plant protein extraction buffer No. 1 (50 mM HEPES-KOH, pH 7.5, 5 mM MgCl2, 300 mM NaCl, 10% glycerol, 2 mM β-Mercaptoethanol, plant protease inhibitor mixture (Sigma, St. Louis, MO), and 1 mM PMSF).

To partition the proteins, the extracts were centrifuged at 100,000 x g for 60 minutes at 4°C. The resulting supernatants were collected for further analysis (cytoplasmic protein). The insoluble pellet was incubated on ice for 30 minutes in the same volume of the collected supernatants (less than 900 µL) of plant extraction buffer No. 2, which contained 1% Triton X-100. Solubilized extracts were centrifuged again at 80,000 x *g* for 60 minutes at 4°C to separate the Triton X-100-soluble and insoluble membrane fractions (TSM and TIM). The resulting supernatant was collected for further analysis (Triton X-100-soluble membrane proteins (TSM)). The pellet was solubilized in the same volume of the collected supernatants (less than 900 µL) of plant extraction buffer No. 3, which contained 1% Triton X-100 and 0.5% SDS. Extracts were centrifuged again at 15,000 x *g* for 30 minutes at 4°C. Supernatants containing TIM fractions were collected for further analysis.

**GC-MS analysis.** Lipid analysis using GC-MS was conducted as previously described [19]. Twenty-five µg of His_6_-GFP-ROP6 enriched or control mick fractions were utilized for the analysis.

**Protein quantifications**. Proteins concentration was determined using BCA reagent and observation at 280 nm. BCA analysis was done using BCA Protein Assay kit (Pierce, Rockford, IL 61105 USA) according to the manufacturer protocol.

Absorbance Assay (280 nm) was done according to [24]. The extinction coefficients were: His_6_-GFP-ROP6 s – 49320 M^-1^ cm^-1^.

**Multiple sequence alignment.** Protein blast (http://blast.ncbi.nlm.nih.gov/Blast.cgi) was done using the Swissprot database. Multiple Sequence Alignment (MSA) was done using MAFFT (http://mafft.cbrc.jp/alignment/server). The results were processed with BioEdit 7.1.3.

**Structural analysis.** For homology modeling, the NEST program [25] was utilized. Nest requires as input a sequence aligned to a structural template. Our structural template was the three-dimensional structure of ROP9 (PDB code 2J07) [26]. The ROP10 sequence was aligned with ROP9. The model was built using default parameters and underwent several rounds of energy minimization. In general, the model appears to fit well onto the surface of ROP9 with few steric clashes. Superposition and RMSD calculation of ROP6 and ROP10, based on the three-dimensional structure of ROP9 (PDB code 2J07) [26], were performed using PyMol (http://pymol.sourceforge.net) with default parameters. The RMSD calculation was 0.247Å and included 127Cα.The EBI-PISA server [27] was used for the identification of the interfacing residues in order to assess the residues required for the protein hydrolysis.

**Light and confocal microscopy.** Brightfield and Nomarsky/differential interference contrast imaging was performed with an Axioplan-2 Imaging microscope (Carl Zeiss, Jena, Germany) using either 20x dry, 40x oil or 63x water immersion objectives with numerical aperture (NA) values of 0.5, 0.9 and 1.2, respectively. Laser scanning confocal microscopy imaging was performed using Leica TCS-SL confocal laser scanning microscope with 20x multi-immersion, 63x water with NAs of 0.7 and 1.2, respectively. Visualization of GFP and Propidium iodide (PI) was carried out using sequential imaging to avoid channel bleed. GFP fluorescence was visualized by excitation with an argon laser at 488 nm. Emission was detected with a spectral detector set between 505 and 530 nm. Propidium iodide (PI) was visualized by excitation with an argon laser set to 514 nm. Emission was detected with a spectral detector set between 600 and 660 nm. Image analysis was carried out with Leica TCS, Image J and Adobe Photoshop. Quantifications of signal intensities were performed with Image J. in the following manner. In 8 bit/pixel scans, the signal intensity on the photomultiplier tubes was converted by the computer to a value on a dimensionless 0–256 scale, where 0 represents no signal and 256 stands for maximal intensity.

**FRAP beam size analysis.** FRAP beam size analysis was carried out as previously described [18,20,22,28,29]. FRAP measurements were carried out at 22°C on the abaxial side of cotyledons, taken from 7 day-old plants. The monitoring Argon ion laser beam (488 nm, 1.2 μW) was focused through the microscope (AxioImager.D1, Carl Zeiss MicroImaging) to a spot with a Gaussian radius of ω = 0.77 ± 0.03 μm using a 63x/1.4 numerical aperture oil-immersion objective (smaller beam size). For studies with a larger Gaussian beam, we used a 40x/1.2 NA water immersion objective, yielding a Gaussian radius of ω = 1.17 ± 0.05 μm. Under these conditions, the ratio between the areas bleached by the larger and smaller beams, ω^2^(40x)/ω^2^(63x), was 2.28 ± 0.15 (n = 59; SD of the ratios was calculated from the bootstrap analysis values as described below). After a brief measurement of the fluorescence intensity with a low-intensity monitoring beam (488 nm, 1 μW), a 5 mW pulse (4–6 ms or 10–20 ms for the 63x and 40x objectives, respectively) bleached 50–70% of the fluorescence in the illuminated spot. Fluorescence recovery was followed by the monitoring beam. The characteristic fluorescence recovery time (τ) and the mobile fraction (R_*f*_) were derived from the FRAP curves by nonlinear regression analysis, fitting to a lateral diffusion process with a single τ value [20,29]. The significance of differences between τ values measured with the same laser beam size was evaluated by one-way ANOVA and Tukey’s post hoc test. To compare between measurements of the τ(40x)/τ(63x) ratio and the ω^2^(40x)/ω^2^ (63x) beam size ratio, we employed two-tailed Student’s *t*-test using bootstrap analysis with 1000 bootstrap samples [30], which is preferable for comparison between ratio values [20,29].

**Plant growth.** Growth of *Arabidopsis* was carried out as previously described [22,31].

***Arabidopsis* transformation.** Transformation of *Arabidopsis* was carried out using the floral dip method [32].

**Plasmolysis.** Ten days old epidermal cotyledons were submerged in 0.8M NaCl solution for 1 minute and then mounted on microscope slides in the same solution.

**Calculation of pavement cell lobe number and circularity.** The determination of cell circularity and lobe count was conducted in accordance with the previously described methods [18]. Specifically, the lobe count was quantified using the skeleton plug-in of Image J. Circularity was calculated using the circularity plug-in of Image J. The analysis was conducted as follows: The abaxial side of seven cotyledons from 10-day-old DAG plants was analyzed (50 cells). Images were captured using a light microscope with a magnification of 20x. Subsequently, each image was used to randomly select seven cells. The cell shape was delineated using the polygon tool and subsequently transferred to an 8-bit new image, where circularity or the skeleton plug-in was applied.

**Statistical analyses.** All data were derived from at least 3 independent experiments and are presented as means ± SD unless otherwise noted in the figure legend. To evaluate the significance of differences between the values of Lobe numbers and circularity, the samples were analyzed by one-way ANOVA followed by Tukey HSD test. This test was also used for the significance of differences between sets of τ or R_*f*_ values in the FRAP studies. All statistical analyses were carried out using Prism10 (GraphPhad, San Diego, CA). FRAP beam-size analysis of the ratio between the τ(40x)/τ(63x(values and the beam size ratio [ω^2^(40x)/ω^2^(63x) employed Bootstrap analysis, as described previously [22,33]. All attempts at replication were successful with similar results.

## Results

### Structure of ROP10 and the position of the cysteine residues

**Fig 1:**
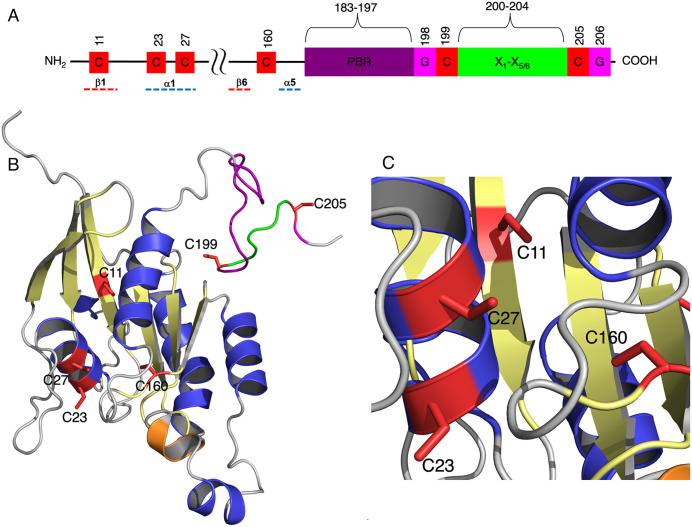
Structure of ROP10. **A)** A schematic 2D model of ROP10, depicting the G domain, hypervariable domain cysteine residues, polybasic region (PBR), and C-terminal GC-CG box. **B)** A 3D model of ROP10, emphasizing the cysteines in the G and hypervariable domains. **C)** A close-up 3D model of the G-domain cysteine residues.

ROP10 contains six cysteine residues: two located in the C-terminal GC-CG box of the hypervariable domain at positions 199 and 205, and four within the G-domain at positions 11, 23, 27, and 160 (Fig 1A). A three-dimensional model of ROP10 indicates that C199 and C205 lie within a flexible loop. The G-domain cysteines C11, C23, and C27 are situated on β-sheet 1 and α-helix 1, while C160 is also found within a flexible loop (Fig 1A to 1C, Fig S1, Fig S2 in S1 File Supporting Information). An overlay of the GDP-bound ROP6 and ROP10 3D models reveals that their overall structures are highly similar, with a root mean square deviation (RMSD) of 0.247 Å. This suggests that functional differences between the two proteins are likely due to variations in the composition of their hypervariable domains and specific residues (Fig S3A in S1 File Supporting Information). The models further show that in ROP6, C21 and C158, and in ROP10, C23 and C160, are located near the GDP/GTP binding site, although they do not act as ligands for the guanine nucleotide (Fig S3B in S1 File Supporting Information). Consistent with this, we previously demonstrated that GTP binding and hydrolysis in the rop6^C21S C58S^ mutant are comparable to WT ROP6 [18]. Given the structural similarity between ROP6 and ROP10, it is likely that the C23S or C160S mutants also retain GTP binding and hydrolysis activity.

### Analysis of ROP10 S-acylation

To determine whether ROP10 is S-acylated *in vivo*, the His_6_-GFP-ROP10 fusion protein was purified from transgenic *Arabidopsis* plants. The acyl lipids were extracted and analyzed by GC-MS [13,14,18,19,34,35].

**Fig 2:**
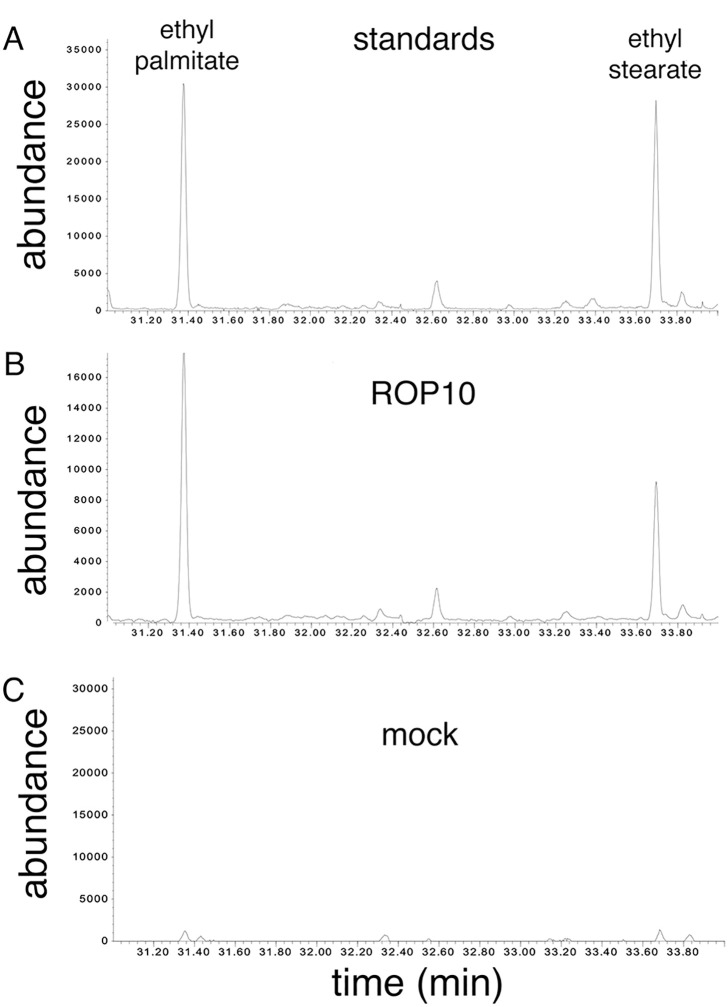
Evidence for S-acylation of ROP10 by palmitic and stearic acids. **A)** Initial standards of ethyl palmitate (retention time: 31 min) and ethyl stearate (retention time: 33 min) derivatives of palmitate and stearate formed by hydrogenation. **B)** ROP10 is S-acylated by both palmitic and stearic acids. **C)** Mock-*Arabidopsis* Col-0 non-transgenic plants protein extract.

Regrettably, affinity purification of the protein on Ni-NTA resin did not yield sufficient quantities of protein to facilitate the analysis of the lipids by GC-MS. To address this limitation, the His_6_-GFP-ROP10 protein was enriched through ion exchange chromatography. The degree of enrichment was evaluated by staining gels with Coomassie blue and identifying the GFP fusion protein by immunoblots decorated with anti-GFP (Fig S4 in S1 File Supporting Information). To ensure that the origin of identified lipids was released from recombinant proteins, the protein purification was also conducted with protein extract from non-recombinant *Arabidopsis* plants (Col-0) (Fig. S4, control in S1 File Supporting Information). During their preparation, the acyl lipid moieties undergo acid esterification and are derivatized to ethyl-palmitate and ethyl-stearate. The ethyl esterification removes the charge from the lipids, which is necessary for their identification by GC-MS [19]. As the ethyl derivatives are formed during sample preparation, we refer to ethyl palmitate as palmitate/palmitic acid and to ethyl stearate as stearate/stearic acid. The GC-MS analysis revealed that ROP10 is very likely S-acylated by palmitic and stearic acids (Fig 2A, Fig 2B). No lipid groups were detected following protein isolation and lipid preparation of the control non-transgenic plants (Fig 2C). Typical GC-MS chromatograms of ethyl palmitate and ethyl stearate are presented in Fig. S5.

The GC-MS analysis is consistent with S-acylation of ROP10 by both palmitic and stearic acids. Since the analysis is based on the release of the lipid moieties from the protein, the GC-MS analysis did not permit the assessment of whether the G domain cysteine residues are also S-acylated. Unfortunately, the purification of the His_6_-GFP-rop10^C199S C205S^ was unsuccessful, possibly due to the protein’s instability.

### Distribution of GFP-ROP10, GFP-rop10CA and their hypervariable and G domains mutants in stably expressing *Arabidopsis*

To evaluate the distribution of ROP10 and its hypervariable domain mutants in *Arabidopsis*, we established transgenic plants that stably express GFP-ROP10, GFP-rop10^Δ193-197^ (lacking the polybasic domain), GFP-rop10^Δ200-204^ (lacking the GC-CG motif residues between C199 and C200), and GFP-rop10^C199 C205S^. The subcellular localization of the examined proteins was determined by co-labeling with propidium iodide (PI) which labels cell wall pectin. Membrane localization was further confirmed by plasmolysis, which induces separation of the plasma membrane from the cell wall, leading to the shrinking of the cell and, consequently, the condensation of the cytoplasm.

**Fig 3:**
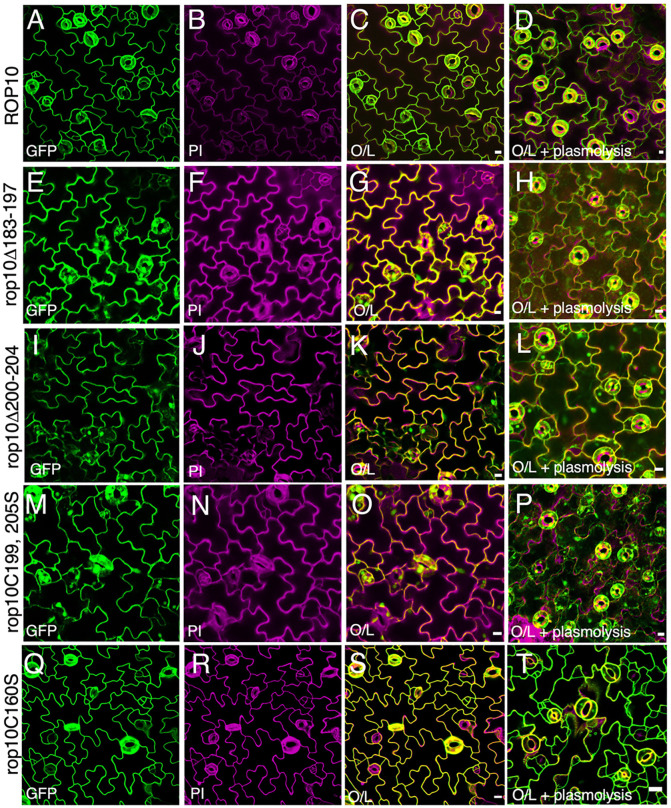
The subcellular localization of ROP10 in stably expressing *Arabidopsis* plants is contingent upon the integrity of its hypervariable domain. **A-T)** Maximum projection confocal images of His_6_-GFP-ROP10 and mutants. Green (GFP), magenta (PI), and overlay of GFP and PI signals before (**C**, **G**, **K**, **O**, and **S**) and after plasmolysis (**D**, **H**, **L**, **P**, and **T**). Note the absence of intracellular GFP fluorescence and the detached cell wall-labeled plasma membrane following plasmolysis in ROP10 (**A-D**) and rop10^C160S^ **(Q-T)**. Observe the intracellular fluorescence in rop10^Δ183-197^ (**E**, **G**, and **H**), rop10^Δ200-204^ (**I**, **K**, and **L**), and rop10^C199S C205S^ (**M**, **O**, and **P**) hypervariable domain mutants. Bars are 10 µm.

The colocalization of GFP-ROP10 and PI, along with the detachment of a GFP-labeled (green) plasma membrane following plasmolysis (Fig 3A to Fig 3D) suggests that GFP-ROP10 is localized within the plasma membrane. See also Fig. S6A and B in S1 File supporting Information for increased magnification. Visualizing the cytoplasmic localization in pavement cells presents a challenge due to the proximity of the cytoplasm to the plasma membrane due to the presence of a vacuole. A meticulous comparison of the GFP fluorescence between GFP-ROP10 and its hypervariable domain mutants reveals the presence of thickenings in the cell periphery, intracellular cytoplasmic strands, and nuclei in the mutants. Notably, following plasmolysis, fluorescent patches emerged in the cells, further corroborating the cytoplasmic localization of the rop10^Δ183-197^, rop10^Δ200-204^, and rop10^C199 C205S^ GFP fusion proteins with hypervariable domain mutations (Fig 3E to Fig 3P). See also Fig S6C and D in S1 File supporting Information for increased magnification. Notably, analysis of the GFP-rop10^C160S^ revealed its primary localization within the plasma membrane (Fig 3Q to Fig 3T).

**Fig 4:**
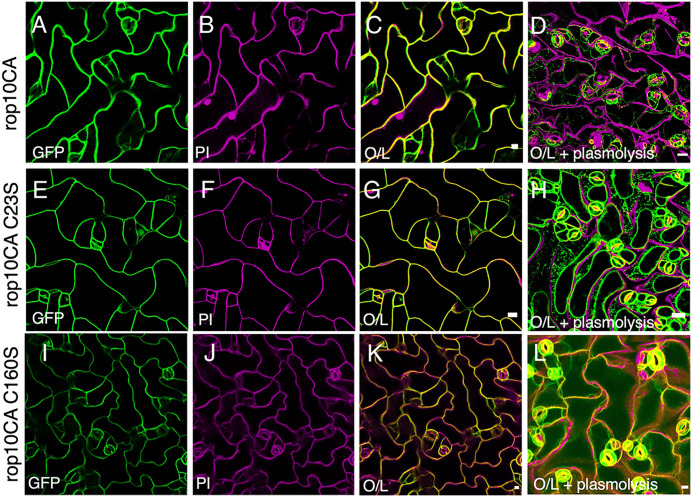
The constitutively active ROP10 and its G domain cysteine mutants are localized in the plasma membrane in stably expressing *Arabidopsis* plants. **A-L)** Maximum projection confocal images of His_6_-GFP-rop10CA and G-domain cysteine mutants. Green (GFP), magenta (PI), and overlay of GFP and PI signals before (**C**, **G** and **K**) and after plasmolysis (**D**, **H** and **L**). Note the absence of intracellular GFP fluorescence in the detached cell wall-labeled plasma membrane following plasmolysis. Observe the changes in cell structure in all the images. Bars are 10 µm.

In the subsequent analysis, we examined the distribution of the constitutively active GFP-rop10CA, GFP-rop10CA^C23S^, and GFP-rop10CA^C160S^ mutants (Fig 4). The colocalization of the GFP and PI labels (Fig 4 C, Fig 4G, Fig 4K), the detachment of GFP-fluorescing membranes from the cell wall following plasmolysis in GFP-rop10CA and the C23S and C160S mutants (Fig 4D, Fig 4H, Fig 4L) indicated that all three proteins were primarily localized in the plasma membrane. See also Fig S6E and F in S1 File supporting Information for increased magnification.

**Fig 5:**
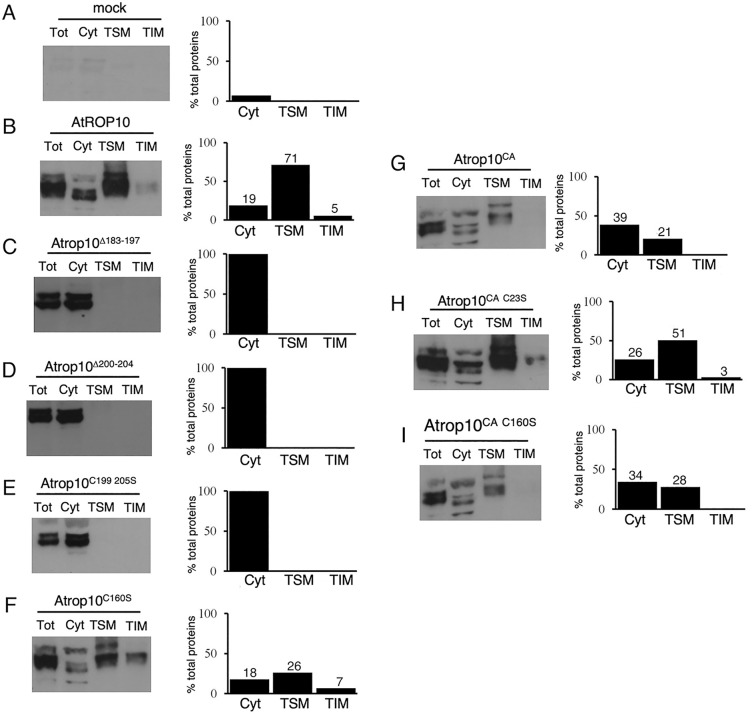
The hypervariable domain, G-domain cysteine C160, and activation status influence the interaction between ROP10 and the plasma membrane. Protein immunoblots, labeled with anti-GFP antibodies, depict the total protein before fractionation (Tot) soluble (Cyt) TSM and TIM membrane fractions of non-transgenic *Arabidopsis* (mock) and transgenic *Arabidopsis* plants expressing His_6_-GFP-ROP10 and rop10 mutants. The bars adjacent to each blot represent the densitometry of the bands in each fraction, calculated as a percentage of the total. See Fig S1 in S2 File Supporting Information – raw images for the full immunoblots.

Further examination of the subcellular localization and membrane compartmentalization of GFP-ROP10 and its mutants was conducted using protein immunoblots (Fig 5, S2 File Supporting Information – raw images). Each panel in Fig 5 presents a protein immunoblot labeled with anti-GFP antibodies, depicting the total protein prior to fractionation (Tot), 100,000 X *g* centrifugation soluble fraction (Cyt), the Triton X-100 soluble 100,000 X *g* pellet fraction (TSM), and the Triton X-100 insoluble 100,000 X *g* pellet fraction (TIM). The bars within each panel represent the densitometry of the blots expressed as percentages relative to the total within each Cyt, TSM, and TIM fraction. The mock immunoblot (Fig 5A, Fig S1 in S2 File Supporting Information – raw images), obtained from non-transgenic *Arabidopsis* plants, serves as the background reference.

The majority of GFP-ROP10 (76%) was detected in the insoluble membrane fraction, primarily (71%) in the TSM. Approximately 20% of the proteins were found in the soluble (Cyt) fraction (Fig 5B). This cytoplasmic fraction was more challenging to detect by fluorescent imaging (Fig 3A to Fig 3D), potentially due to reduced sensitivity or dispersion of the soluble signal in the fluorescent images. The hypervariable domain mutants rop10^Δ182-197^, rop10^Δ200-204^, and rop10^C199S C205S^ were exclusively detected in the soluble (Cyt) fraction (Fig 5C to Fig 5E), consistent with the fluorescent imaging results (Fig 3E to Fig 3P). Only 51% of GFP-rop^C160S^ were recovered in the Cyt, TSM, and TIM fractions (Fig 5F), suggesting that this mutant exhibited reduced stability following cell fractionation. The recovery of GFP-rop10CA following fractionation (*i.e.*, the sum of the protein detected in the Cyt, TSM and TIM) was 60% of the total loaded, with 39% of the protein found in the soluble fraction and 21% in the TSM. Notably, no protein was detected in the TIM fraction (Fig 5G). The results of the immunoblots deviated from the fluorescent imaging, where GFP-rop10CA was detected mainly in the plasma membrane (Fig 4A to Fig 4D). Importantly, the distribution of GFP-rop10CA was significantly different from that of GFP-rop6CA, which accumulated almost exclusively in the TIM fraction and exhibited stable membrane association, whereas GFP-rop10CA displayed reduced membrane affinity [14,18,19].

The findings presented in Fig. 5 and the appearance of multiple bands suggest that GFP-rop10CA exhibited reduced stability following fractionation, leading to membrane detachment and potential degradation or post-translational modifications. Intriguingly, 80% of the GFP-rop10CA^C23S^ mutant was recovered with a larger fraction (51%) in the TSM, 26% in the soluble fraction, and 3% in the TIM (Fig 5H). Thus, the C23S mutation stabilized the CA mutant and its interaction with the membrane. GFP-rop10CA^C160S^ was more stable than rop10CA but less stable than rop10CA^C23S^, with a recovery of 62% of the total protein, and the C160S mutation also appears to enhance the interaction of rop10CA with the membrane. Similar to the rop10CA mutant, it was not detected in the TIM fraction (Fig 5I).

These results are significantly different from the fractionation of GFP-ROP6, which partitioned between TSM and TIM, and GFP-rop6CA, which accumulated in TIM [18]. Additionally, in the background of the CA mutation, the C23S and C160S mutations increased the membrane association and stabilized the protein.

### FRAP beam-size analysis of the interaction dynamics of GFP-ROP10 and its mutants with the plasma membrane

The results of the immunoblot analysis (Fig 5) indicated that GFP-ROP10 mutants with a constitutively activating mutation, especially rop10CA and ropCA^C160S^, exhibit reduced membrane association and stability. This prompted us to investigate the effects of specific mutations on the dynamic interaction of GFP-ROP10 and several mutants (rop10^C160S^, rop10CA, rop10CA^C23S^, and rop10CA^C160S^) with the plasma membrane using FRAP beam-size analysis [20]. This method (see Experimental Procedures) employs two Gaussian laser beam sizes, generated using a 63x (smaller Gaussian radius, ω) or a 40x (larger ω) objectives to focus the laser beam on the plasma membrane. The ratio between the illuminated areas, ω^2^(40x)/ω^2^(63x), was 2.28 ± 0.15. For FRAP by lateral diffusion, τ (*t*_1/2_ for fluorescence recovery) reflects the characteristic diffusion time (τ_*D*_), which is proportional to the area bleached by the laser beam (τ_*D*_ = ω^2^/4*D*; where *D* is the lateral diffusion coefficient). Therefore, for FRAP by lateral diffusion, the τ(40x)/τ(63x) ratio should equal the beam-size ratio (2.28). On the other hand, a τ ratio of 1 is expected for FRAP by exchange between membrane-associated and cytoplasmic pools of the protein, where τ is the characteristic exchange time τ_ex_, which is independent of the beam size. τ ratios in between 2.28 and 1 indicate a mixed recovery mode [20]. Of note, since once a bleached fluorescent molecule is replaced in the bleached area by one process its fluorescence has already been recovered, the faster of the two processes (diffusion *vs*. exchange) dominates the recovery mode. Thus, recovery by pure lateral diffusion indicates that the exchange rate is much slower than the lateral diffusion rate and does not contribute to the measured recovery. On the other hand, an increase in the exchange rate (which is typical of weaker interactions with the membrane) results in a shift of the recovery mode towards exchange (a τ ratio closer to 1) [28].

**Fig 6:**
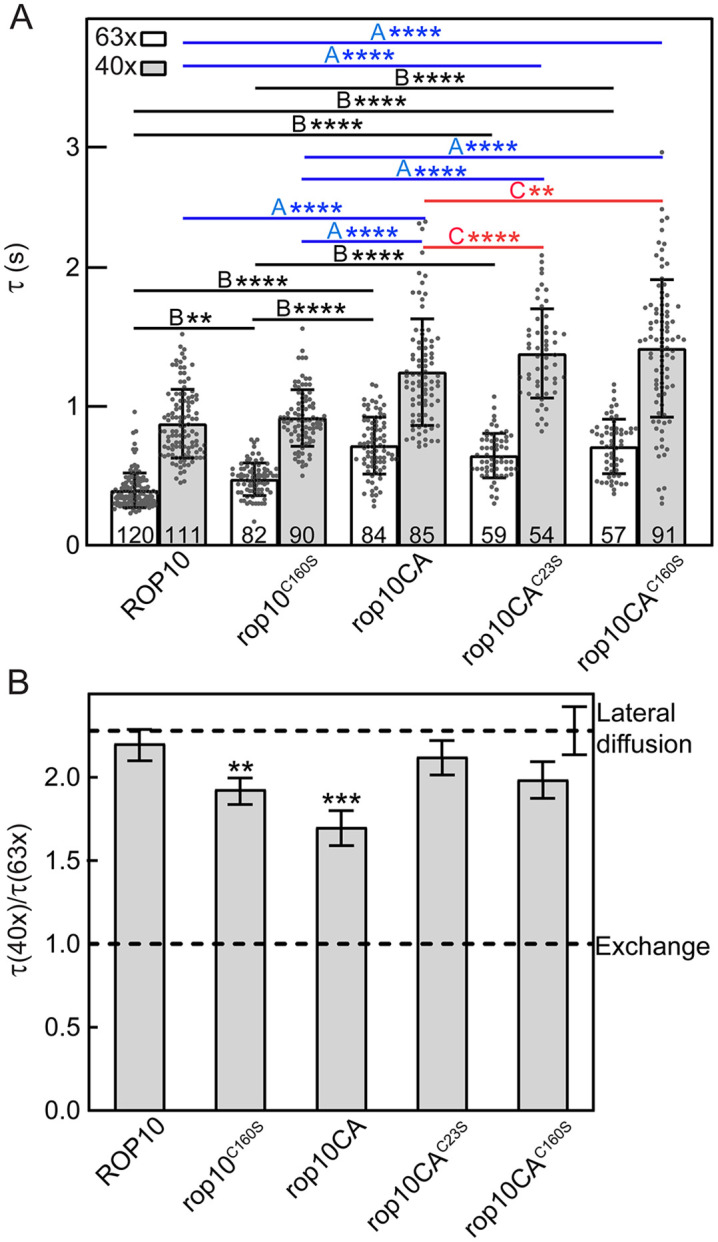
FRAP beam size analysis of the interaction dynamics of ROP10 and rop10 mutants with the membrane. For each ROP10 variant, the specific transgenic lines employed are identical to those designated in the legend to Fig 7. **A)** Average τ values obtained in FRAP measurements (for sample representative FRAP curves, see Fig S6 in S1 File Supporting Information). Bars are means ± SD of multiple independent measurements, each conducted on a different cell (the number of measurements is depicted within each bar). Asterisks indicate significant differences (**, p ≤ 0.01, ****, p ≤ 0.001; one way ANOVA and Tukey’s post-hoc test) between the τ values of the indicated pairs of ROP10 variants, comparing separately the τ values obtained with the 40x objective (B, black lines) and the 63x objective (A, blue lines; **,p ≤ 0.01, ****,p ≤ 0.001 derived by one way ANOVA and Tukey’s post-hoc test). Apart of the differences between ROP10 WT or rop10^C160^ and the mutants, there were significant differences between the τ(63x) values of ROP10CA and the double mutants rop10CA^C23S^ and rop10CA^C160S^ (C, red lines). The mobile fraction (R_*f*_) values did not show significant differences between all ROP10 variants (see Fig S8 in S1 File Supporting Information). **B)** FRAP beam-size bootstrap analysis. The studies employed 40x or 63x objectives, focusing the laser beam to larger (40x) and smaller (63x) Gaussian spots, with a beam size ratio of ω^2^(40x)/ω^2^(63x) = 2.28 ± 0.15 (n = 59 independent measurements) [33]. A τ(40x)/τ(63x) ratio similar to 2.28 is obtained for FRAP by lateral diffusion, while a τ ratio of 1 indicates recovery by exchange. The SD values for the τ ratios and beam-size ratios were calculated from the τ values shown in panel A, using bootstrap analysis (1,000 bootstrap resampling values). While the τ ratio of ROP10 was not significantly different from the 2.28 beam-size ratio, indicating recovery by lateral diffusion, the τ ratios of rop10CA and rop10^C160S^ significantly deviated towards 1, suggesting a significant contribution by exchange to the FRAP. On the other hand, mutation of C160 or C23 on the background of rop10CA (rop10CA^C160S^ and rop10CA^C23S^) returned the τ ratio to values not significantly different from the 2.28 beam-size ratio, indicating that the additional mutations returned the exchange rates to much slower values than the lateral diffusion. Asterisks indicate significant differences between the τ(40x)/τ(63x) ratio of a given ROP10 variant and the 2.28 beam-size ratio (**, p ≤ 0.02; ***, p ≤ 0.002; Student’s two-tailed t-test, comparing each τ ratio to the beam-size ratio).

Typical fluorescence recovery curves obtained for the GFP-ROP10 variants using the smaller beam size (63x objective) are shown in Fig S7 (S1 File Supporting Information), while quantitative data summarizing multiple experiments using the two different Gaussian laser beam sizes are depicted in Fig 6A and Fig S8 (S1 File Supporting Information). The *D* values can be calculated from the τ(63x) values, where the potential contribution of exchange is the lowest as the characteristic time of recovery by diffusion is faster at the smaller beam size. Such calculation from the τ(63x) values shown in Fig 6A yields *D* values of 0.37 and 0.32 μm^2^/s for ROP10 and rop10^C160S^, respectively, and significantly lower values (0.20–0.23 μm^2^/s) for the CA mutants (rop10CA, rop10CA^C23S^, and rop10CA^C160S^). Since the *D* values of ROP10 and rop10^C160S^ are typical of small GTPases in the inner leaflet of the plasma membrane, such as Ras proteins [28,36], the slower diffusion of ROP10 mutants containing the CA mutation suggests that their lateral diffusion is retarded by interactions with slower-diffusing membrane proteins, most likely involving the altered conformation of the activated G domain. These findings are in line with an earlier report on the membrane interactions of H-Ras, where the catalytic domain of the GTP-loaded conformation induced repulsion from the plasma membrane [29]. The mobile fraction values of the fluorescence recovery (R_*f*_) for all the GFP-ROP10 proteins measured were similar (0.80–0.90) and were not significantly different from each other (Fig S8 in S1 File Supporting Information).

Examination of the τ(40x)/τ(63x) ratios of the ROP10 mutants relative to the beam-size ratio (Fig 6B) yields important information on their affinity to the plasma membrane, since weaker interactions can make the exchange rate faster, thus increasing the contribution of exchange to the recovery and inducing a shift from recovery by lateral diffusion towards recovery by exchange. As shown in Fig 6B, the τ(40x)/τ(63x) ratio of ROP10 was indistinguishable from the beam size ratio [ω^2^(40x)/ω^2^(63x)] of 2.28, indicating recovery by pure lateral diffusion (slow exchange rate relative to the diffusion rate) [20,29]. In the rop10^C160S^ mutant, the τ(40x/τ(63x) ratio was significantly reduced below the 2.28 beam-size ratio (to 1.92), indicating faster exchange rate, as expected for weaker membrane interactions. This suggests that the C160 residue, which may undergo transient *S*-acylation as in ROP6 [18], stabilizes the interaction of WT ROP10 with the plasma membrane, a stabilization that is lost in the mutant lacking cysteine in this position. Of note, the τ ratio of the constitutively active rop10CA was dramatically reduced (to 1.7; Fig 6B), demonstrating a high contribution of exchange to the fluorescence recovery and suggesting faster exchange rate and thus weaker affinity to the membrane. This is in line with the significant change in the τ values of this mutant, most likely due to the shift to exchange as the affinity to the membrane decreases. Interestingly, the τ(40x)/τ(63x) ratios of rop10CA^C23S^ and rop10CA^C160S^ (2.12 and 1.98, respectively; Fig 6B) were not significantly lower than the beam-size ratio of the same mutants in the WT ROP6 background, suggesting that on the background of the constitutively active mutant (GTP-bound conformation), the C23 and C160 have a negative contribution to the interactions of ROP10 with the plasma membrane. These results are mainly consistent with those of the studies on the steady state distribution of these ROP10 proteins (WT and mutant) between membrane and cytosolic fractions (Fig 5G to Fig 5I). The difference between rop10CA and rop10CA^C160S^ in Fig 6B, which is not detected in their membrane fractionation (Fig 5), is most likely due to the higher sensitivity of the FRAP beam-size measurements.

### Evaluation of the role of ROP10 in pavement cells: an analysis of cell polarity

ROPs regulate the formation of lobes and indentations during pavement cell growth. Cell circularity is defined as 4π x area/perimeter². Overexpression of constitutively active ROPs results in increased pavement cell circularity and reduced lobe number per cell, making them useful for assessing ROP function. Cell circularity and lobe number are inversely correlated with R² = 0.975. The average circularity and lobe number of Col-0 WT plants are 0.213 and 9, respectively, while the average circularity and lobe number of *rop10CA* cells are 0.44 and 3, respectively (Fig S9 and Fig S10 in S1 File Supporting Information). See representative images in Fig 4 and Fig 5.

**Fig 7:**
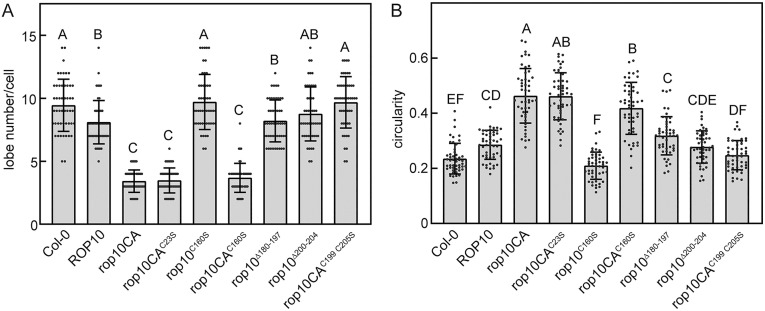
The intact hypervariable domain and G domain cysteine C160 are crucial for ROP10’s influence on cellular structure. **A)** lobe number and **B)** circularity. Error bars – SD. Letters above bars in all correspond to statistically significant difference (one-way ANOVA and Tukey’s HSD test, p ≤ 0.05). Representative images are show in Figs 3 and 4. Fifty cells were measured for each cell line. For each ROP10 variant, two cell lines were generated and tested. The results shown here are for one line expressing the variant, as both lines gave identical results (Fig S10 in S1 File).

Figs 7 and S10 in S1 File Supporting Information present the analysis of lobe number and circularity in WT Col-0 and the different ROP10 WT and mutant transgenic genotypes (representative images are shown in Fig 3 and Fig 4). Overexpression of ROP10 resulted in a significant reduction in the number of lobes and increased circularity. However, the lobe number and circularity of rop10^C160S^ plants were not significantly different from that of WT Col-0, suggesting that C160 is critical for ROP10 function. Suppression of ROP10 membrane attachment by hypervariable domain mutations C199 and C205, compromised the effect of ROP10 on cell polarity, as indicated by lobe number and circularity, which were not significantly different compared to WT Col-0.

Significantly, immunoblot and FRAP beam-size analyses demonstrated increased plasma membrane interaction and stability of the C23S and C160S mutants in the rop10CA background (Figs 5 and 6), suggesting that, in constitutively active mutants, membrane dissociation is decoupled from its effects on pavement cell phenotype.

Mutations in rop6CA (C21S and C158S) result in decreased circularity and increased lobe number compared to rop6CA [18]. Therefore, the analysis of pavement cell polarity of rop10CA mutants indicates differential modes of plasma membrane interaction and the function of C23 and C160.

## Discussion

In contrast to fungi and animal cells, plants possess only a single family of Rho GTPases, namely the ROPs. Notably, type-II ROPs emerged in seed plants due to the insertion of an intron at the 3’ end of an ancestral *ROP* gene [3]. Studies conducted in *Arabidopsis* and tomato have suggested that type-II ROPs may possess unique functions [4,11,22,37]. Furthermore, it has been demonstrated that type-II ROPs do not interact with RhoGDI, implying a distinct regulatory mechanism compared to type-I ROPs [22]. Yet, prior to this research, direct *in vivo* evidence for numerous characteristics of type-II ROPs (S-acylation, the identities of the acyl moieties, membrane distribution, membrane interaction dynamics, and the relationship between membrane association and function) was lacking.

Our results suggest that ROP10 is S-acylated in *Arabidopsis* by both palmitic and stearic acids. Additionally, its membrane association is less stable compared to that of type-I ROPs, and it predominantly accumulates in Triton X-100 soluble membrane. The membrane interaction studies and functional assays presented here indicate that C160 is indispensable for both stable membrane association and ROP10 function. Structural analysis reveals that C160 is situated within a flexible loop, and the structures of ROP10 and ROP6 exhibit remarkable similarity. Notably, C158, the orthologous cysteine residue in ROP6, is transiently S-acylated and is also localized within a flexible loop. Considering the structural congruence between ROP6 and ROP10, and the pivotal role of ROP10 C160 in stabilizing membrane association, it is plausible that ROP10 is S-acylated on C160. Given the limitations of GC-MS analysis, future work using alternative approaches, such as acyl-biotin exchange in combination with site-directed mutagenesis and functional assays, will be required to map the specific S-acylation sites in ROP10 and other type-II ROPs.

The relatively low levels of GFP-rop10^C160S^ and GFP-rop10CA in the immunoblot analysis (Fig 5) likely reflects their weaker membrane association, resulting in reduced stability. This notion was corroborated by the FRAP beam size analysis studies, which demonstrated reduced affinity to the membrane which leads to faster membrane-cytoplasmic exchange rates of both proteins (Fig 6B). However, these differences between the membrane localization of GFP-ROP10, rop10^C160S^ and rop10CA were difficult to visualize by confocal imaging (Fig 3 and Fig 4), possibly due to sensitivity of the method. These findings suggest that steady-state imaging may not accurately reflect the dynamic protein-membrane association and, consequently, does not provide a comprehensive understanding of the membrane interaction of membrane-associated proteins and their sub-compartmentalization.

The diminished membrane association of GFP-rop10^C160S^ (Fig 6B) and its reduced gain of function effect on cell polarity (Fig 7) establish a causal link between the stability of the membrane association of ROP10 and its function. Notably, circularity and lobe number in rop10CA^C23S^ and rop10CA^C160S^ were not significantly different compared to rop10CA. Furthermore, in the rop10CA background, the loss of the cysteine in the C23S and C160S mutants stabilized their interaction with the membrane (Fig 5, and the reduced contribution of exchange to the fluorescence recovery of these mutants relative to rop10CA; Fig 6B). This observation is in contrast with earlier findings on rop6CA, whose membrane association was weaker in the C21S and C160S mutants, which also had a reduced effect on cell polarity [18].

Both type-I and type-II ROPs bind to the inner leaflet of the plasma membrane through their lipid modifications and interaction of positively charged lysine and arginine residues in their PBR domain with anionic phospholipids. However, the reasons for their distinct membrane interaction dynamics and partitioning remain unresolved. It has been proposed that prenyl groups (geranylgeranyl or farnesyl) of prenylated ROPs bind to hydrophobic pockets within membrane proteins, enhancing their association with the plasma membrane [38]. Type-I ROPs are primarily geranylgeranylated [13] and interact with RhoGDI. *In vitro* assays demonstrated that RhoGDI can extract type-I ROPs from the plasma membrane, but does not bind to type-II ROPs and does not affect their membrane association [22]. Possibly, type-I ROPs interact with additional membrane components through their geranylgeranyl group, and their release from the plasma membrane requires RhoGDI. On the other hand, type-II ROPs are usually modified by saturated fatty acids such as palmitic and stearic acids *via* S-acylation of cysteine residues, and are likely embedded directly within the membrane, potentially exhibiting weaker membrane interaction relative to type-I ROPs. The reduced membrane association of rop10CA compared to wild-type ROP10 may result from weakened interactions between its S-acyl lipid anchors and plasma membrane lipids, potentially due to conformational changes induced by GTP binding. In contrast, in type-I ROPs, the geranylgeranyl moiety may maintain stable interactions with membrane components even in the GTP-bound state, thereby supporting stronger membrane association upon activation.

FRAP beam size analysis revealed that coexpression of ROP11, a type-II ROP, with ROPGEF and ROPGAP leads to its immobilization (strong reduction in its mobile fraction, where the remaining recovery is mostly by exchange) [22]. As ROP11 domains are involved in the formation of secondary cell wall pits in the metaxylem [37], it is possible that the altered membrane interaction dynamics of ROP11 are essential for pit formation at multiple positions along the metaxylem cells. In addition, root hairs develop through tip growth, which is associated with accumulation of ROP2 at the tip of the growing root hair [39]. RhoGDI1 mutants exhibit split root hairs with multiple tips, and ROP2 distribution is dispersed [40]. Stable tip localization of ROP2 may be required during root hair growth, in which case the weaker association of type-II ROPs with the membrane may not support sustained tip growth.

In summary, the findings reported here suggest that type-II Rho GTPases operate from distinct plasma membrane compartments and exhibit unique interaction dynamics with the membrane. The reduced membrane affinity of the GTP-bound active form, together with the reported lack of interaction with RhoGDI, may confer increased mobility and facilitate rapid remodeling or redistribution within membrane domains. Such dynamic behavior could be particularly important in developmental contexts that require the coordinated patterning of multiple sites, such as metaxylem pit formation, where localized and transient signaling events are essential. Therefore, although seed plants do not possess distinct Rho, Rac, and Cdc42 subfamilies, the differential membrane interaction properties of type-I and type-II ROPs may represent a mechanism for functional diversification within the ROP family.

## Supporting information

S1 File Supplementary Tables and Figures. (PDF)

S2 File Raw Images. (PDF)
