# Supplementary material for: C-terminal S-acylation governs membrane distribution, interaction dynamics and function of a plant Rho GTPase
Source: PLoS One. 2026 Apr 30;21(4):e0348444. doi: 10.1371/journal.pone.0348444 (PMC13132179; doi:10.1371/journal.pone.0348444)
Supplement: S1 File — (PDF) [file pone.0348444.s001.pdf]

S1 Supporting Information

C-terminal S-acylation governs membrane distribution, interaction dynamics and function of a plant Rho GTPase

Amir Akerman, Orit Gutman, Keren E. Shapira, Efraim Lewinsohn, Yoav I. Henis and Shaul Yalovsky

This file includes 4 supplementary tables, 10 supplementary figures (Figs S1 to Fig S10).

Supplementary Tables

Table S1: List of plasmids used in this study

Plasmid name	Description	Antibiotics	Source
pCAMBIA 2300	Plant TDNA-based binary vector, *35S* promoter and NOS 3'-end,	Kan	Cambia
pCAMBIA 3300	Plant TDNA-based binary vector, *35S* promoter and NOS 3'-end,	BAR	Cambia
pDoner221	pDoner221	Kan	Gateway, Invitrogen
pENTR1A	pENTR1A	Kan	Gateway, Invitrogen
pEarley100	A Gateway compatible plant binary vector	Kan	[1]
pJet1.2	pJet1.2 cloning plasmid	Amp	Fermentas
pRTL Hisx6 GFP	Based on the pGFP-MRC 35S::GFP	Amp	[2]
pSY108	pRTL GFP-ROP10	Amp	Lab stocks
pSY123	pRTL GFP-rop10$^{C199S+205S}$	Amp	Lab stocks
pSY173	pRTL GFP-rop10CA^{G15V}	Amp	Lab stocks
pSY196	pRTL GFP-rop10$^{\Delta 200-204}$	Amp	Lab stocks
pSY444	pRTL GFP-rop10DN^{D124A}	Amp	Lab stocks
pSY1768	pRTL GFP-rop10$^{\Delta 183-197}$	Amp	Lab stocks
pSY2100	pDoner_R221_HIS$_6$	Kan	This study
pSY2101	pEntr_P3_ROP10_CDS	Kan	This study
pSY2102	pDoner_221_attB1_ROP10-CDS_attB2	Kan	This study
pSY2103	pDoner_P2RP3_ROP10_CDS	Kan	This study
pSY2105	pRTL His$_6$-GFP-ROP10	Amp	This study
pSY2106	pRTL His$_6$-GFP-rop10CA^{G15V}	Amp	This study
pSY2107	pRTL His$_6$-GFP-rop10DN^{D124A}	Amp	This study
pSY2108	pCAMBIA2300 His$_6$-GFP-ROP10	Kan	This study
pSY2109	pCAMBIA2300 His$_6$-GFP-rop10CA^{G15V}	Kan	This study
pSY2110	pCAMBIA2300 His$_6$-GFP-rop10DN^{D124A}	Kan	This study
pSY2112	pNTAPa_ROP10	Spec	This study
pSY2117	pEntr1A_MIRI_ROP10-11	Kan	This study
pSY2118	pEntr1A_MIRII_ROP10-11	Kan	This study
pSY2119	pEearly100_MIRI_ROP10-11	Kan	This study
pSY2120	p Eearly100_MIRII_ROP10-11	Kan	This study
pSY2125	pRTL His$_6$-GFP-rop10$^{C199S\ C205S}$	Amp	This study
pSY2126	pCAMBIA2300 His$_6$-GFP-rop10$^{C199S\ C205S}$	Kan	This study
pSY2128	pRTL His$_6$-GFP-rop10$^{\Delta 200-204}$	Amp	This study
pSY2129	pRTL His$_6$-GFP-rop10$^{\Delta 183-197}$	Amp	This study
pSY2130	pCAMBIA2300 His$_6$-GFP-rop10$^{\Delta 200-204}$	Kan	This study
pSY2131	pCAMBIA2300 His$_6$-GFP-rop10$^{\Delta 183-197}$	Kan	This study
pSY2138	pRTL His$_6$-GFP-ROP10CA^{C160S}	Amp	This study
pSY2139	pCAMBIA2300 His$_6$-GFP-rop10CA^{C160S}	Kan	This study
pSY2143	pJet1.2 35S His$_6$-GFP-rop10CA^{C23S}	Amp	This study
pSY2144	pCAMBIA2300 His$_6$-GFP-rop10CA^{C23S}	Kan	This study
pSY2146	pCAMBIA3300 His$_6$-GFP-ROP10WT	Kan	This study

pSY2147	pCAMBIA3300 His_6-GFP-rop10CA	Kan	This study
pSY2148	pCAMBIA3300 His_6-GFP-rop10CA^{C160S}	Kan	This study
pSY2149	pCAMBIA3300 His_6-GFP-rop10$^{C199S\ C205S}$	Kan	This study
pSY2150	pRTL His_6-GFP-rop10^{C160S}	Amp	This study
pSY2155	pCAMBIA3300 His_6-GFP-rop10^{C160S}	Kan	This study

Table S2: List of bacterial strains used in this study

Bacterial name	Description	Antibiotics	Source
SY2100	DH5α/pDoner_R221__6xHIS	Kan	This study
SY2102	DH5α/pDoner_221_attB1_ROP10-CDS_attB2	Kan	This study
SY2103	DH5α/pDoner_P2RP3_ROP10_CDS	Kan	This study
SY2104	DH5α/pB7_35_His$_6$_ROP10_CDS	Spec	This study
SY2105	DH5α/pRTL His$_6$-GFP-ROP10	Amp	This study
SY2106	DH5α/pRTL His$_6$-GFP-rop10CA^{G15V}	Amp	This study
SY2107	DH5α/pRTL His$_6$-GFP-rop10DN^{D124A}	Amp	This study
SY2108	DH5α/pCAMBIA2300 His$_6$-GFP-ROP10	Kan	This study
SY2109	DH5α/pCAMBIA2300 His$_6$-GFP-rop10CA^{G15V}	Kan	This study
SY2110	DH5α/pCAMBIA2300 His$_6$-GFP-rop10DN^{D124A}	Kan	This study
SY2111	GV3101/pCAMBIA2300 His$_6$-GFP-ROP10	Kan, Gent	This study
SY2112	DH5α/pNTAPa_ROP10 new	Spec	This study
SY2113	GV3101/pNTAPa_ROP10	Spec, Gent	This study
SY2114	GV3101/pCAMBIA2300 His$_6$-GFP-rop10CA^{G15V}	Kan, Gent	This study
SY2115	GV3101/pCAMBIA2300 His$_6$-GFP-rop10DN^{D124A}	Kan, Gent	This study
SY2116	DH5α/pj201:29368_MIRII_ROP10-11	Kan	This study
SY2117	DH5α/pEntr1A_MIRI_ROP10-11	Kan	This study
SY2118	DH5α/pEntr1A_MIRII_ROP10-11	Kan	This study
SY2119	DH5α/pEearly100_MIRI_ROP10-11	Kan	This study
SY2120	DH5α/p Eearly100_MIRII_ROP10-11	Kan	This study
SY2123	GV3101/pEARLY100_MIR_I_ ROP10-11	Kan, Gent	This study
SY2124	GV3101/pEARLY100_MIR_II_ ROP10-11	Kan, Gent	This study
SY2125	DH5α/pRTL His$_6$-GFP-rop10$^{C199S\ C205S}$	Amp	This study
SY2126	DH5α/pCAMBIA2300 His$_6$-GFP-rop10$^{C199S\ C205S}$	Kan	This study
SY2127	GV3101/pCAMBIA2300 His$_6$-GFP- rop10$^{C199S\ C205S}$	Kan, Gent	This study
SY2128	DH5α/pRTL His$_6$-GFP-rop10 $^{\Delta200\text{-}204}$	Amp	This study
SY2129	DH5α/pRTL His$_6$-GFP-rop10 $^{\Delta183\text{-}197}$	Amp	This study
SY2130	DH5α/pCAMBIA2300 His$_6$-GFP-rop10$^{\Delta200\text{-}204}$	Kan	This study
SY2131	DH5α/p CAMBIA2300 His$_6$-GFP-rop10$^{\Delta183\text{-}197}$	Kan	This study
SY2132	GV3101/pCAMBIA2300 His$_6$-GFP-rop10$^{\Delta200\text{-}204}$	Kan, Gent	This study
SY2133	GV3101/pCAMBIA2300 His$_6$-GFP-rop10$^{\Delta183\text{-}197}$	Kan, Gent	This study
SY2138	DH5α/pRTL His$_6$-GFP-rop10CA^{C160S}	Amp	This study
SY2139	DH5α/pCAMBIA2300 His$_6$-GFP-rop10CA^{C160S}	Kan	This study
SY2140	GV3101/pCAMBIA2300 His$_6$-GFP-rop10CA^{C160S}	Kan, Gent	This study
SY2143	DH5α/pJet1.2 35S His$_6$-GFP-rop10CA^{C23S}	Amp	This study
SY2144	DH5α/pCAMBIA2300 His$_6$-GFP-rop10CA^{C23S}	Kan	This study
SY2145	GV3101/pCAMBIA2300 His$_6$-GFP-rop10CA^{C23S}	Kan, Gent	This study
SY2146	DH5α/pCAMBIA3300 His$_6$-GFP-ROP10WT	Kan	This study
SY2147	DH5α/pCAMBIA3300 His$_6$-GFP-rop10CA^{G15V}	Kan	This study
SY2148	DH5α/pCAMBIA3300 His$_6$-GFP-rop10CA^{C160S}	Kan	This study
SY2149	DH5α/pCAMBIA3300 His$_6$-GFP-rop10$^{C199S\ C205S}$	Kan	This study
SY2150	DH5α/pRTL His$_6$-GFP-rop10^{C160S}	Amp	This study
SY2151	GV3101/pCAMBIA3300 His$_6$-GFP-ROP10WT	Kan, Gent	This study
SY2152	GV3101/pCAMBIA3300 His$_6$-GFP-rop10CA^{G15V}	Kan, Gent	This study
SY2153	GV3101/pCAMBIA3300 His$_6$-GFP-rop10$^{C199S\ C205S}$	Kan, Gent	This study
SY2155	DH5α/pCAMBIA3300 His$_6$-GFP-rop10^{C160S}	Kan	This study
SY2156	GV3101/pCAMBIA3300 His$_6$-GFP-rop10^{C160S}	Kan, Gent	This study

Table S3: List of oligonucleotide primers used in this study

Primers Name	description	F/R	Sequence 5'-3'	Target gene
SYP2100	HISx6_attB1_F	F	GGGGACAAGTTTGTACAAAAAAGCAGGCTTAATGCATCATCATCATCATCATCACCC	ROP10
SYP2101	HISx6_attB2_R	R	GGGGACCACTTTGTACAAGAAAGCTGGGTCATGATGATGATGATGATGATGGCTTAAGCC	ROP10
SYP2102	attB1_ROP10_CDS_F1	F	AAAAAAGCAGGCTTACAAGGAGGAGGAGGAGGCTCCGAAACCATGTCCATCATCATCATCATCATCATCATC	ROP10
SYP2103	attB1_ROP10_CDS_F2	F	GGGGACAAGTTTGTACAAAAAAGCAGGCTTAGGGAAGGCTCCGAAACCATG	ROP10
SYP2106	attB2_ROP10_CDS_R1	R	CAAGCAAGCTGGGTTAATCTTCCGGCAGAAACCATGTGCCCAGAAGTTGTTC	ROP10
SYP2107	attB2_ROP10_CDS_R2	R	TATAATAAAGTTGCTCAATTCTCCCAAGTCGCATTTCTTCCTCCCCTTTTC	ROP10
SYP2110	attB3_ROP10_R1	F	GGGGACAACTTTGTACAATAAAGTTGCTCCATACTTCCATAAACCCTTACCT	ROP10
SYP2111	attB3_ROP10_R2	R	TATAATAAAGTTGCTCAATCCTGATTCCCAGTCGCATTTGTTCGCTTCAT	ROP10
SYP2112	attB4_ROP10_F1	F	FAGAAAAGTTGACTTCCACCAGGATTTTCCTGATCCTGATCCTGA	ROP10
SYP2113	attB4_ROP10_F2	F	GGGGACAACTTTGTACAAAAAGTTGCAACTTGCCCTTCCCCAGA	ROP10
SYP2114	TEST_promotor_ROP10_F500	F	TGTTCTACAGGTTCTTCCATTTCCAGTTTGGGTCTCTCCAGA	ROP10
SYP2115	TEST_promotor_ROP10_P991	R	GGGGACAACTTTGTACAATAAAGTTGCCTTTGCTGCCAAGTTCTTCC	ROP10
SYP2116	TEST_promotor_ROP10_F1499	F	GGGGACAACTTTGTACAATAAAGTTGCTTTGGTCAAATCATCTGTGAAAAG	ROP10
SYP2117	TEST_promotor_ROP10_P1991	R	TATAATAAAGTTGCTCAATGCTCCAGTTGGTTCCCAGTCCTTTCC	ROP10
SYP2118	TEST_promotor_ROP10_F2501	F	GGGGACAACTTTGTACAATAAAGTTGCTTTCTTACCATTCCTTCT	ROP10
SYP2119	TEST_promotor_ROP10_F3011	R	TATAATAAAGTTGCTCAATGCTCCTCCATTGGTTCCAGAT	ROP10
SYP2120	attB3_ROP10_CDS_R1	F	GGGGACAACTTTGTACAATAAAGTTGCTCCATACTTCCATAAACCCTTACCT	ROP10
SYP2121	attB3_ROP10_CDS_R2	R	TATAATAAAGTTGCTCAATTCTTCCCACCAGAATTTCTTCC	ROP10
SYP2122	TEST_attB2_ROP10_attB3_F501	F	GGGGTTGAAGCCATCGCCACCCAAGTTGCCTCAATCCTTCC	ROP10
SYP2123	TEST_attB2_ROP10_attB3_F983	F	CCCAATTGTGCTTCTGCT	ROP10
SYP2124	TEST_attB2_ROP10_attB3_F1485	F	CCCGCAGTGAAACAAAAGG	ROP10
SYP2125	TEST_attB2_ROP10_attB3_F1953	F	GTGGTTAAGCCACTTTATTATTATCCAAGATTAAGAT	ROP10
SYP2126	attB4_35S_Omega_attB1_R1	R	TTCACAAACTTGAAATTGTAAATAGTAATTGTAATGTTGTTTG	ROP10
SYP2127	attB4_35S_Omega_attB1_R2	R	GGGGACTGCTTTTTTGTACAAACTTGAAATTGTAAATAGTAA	ROP10

SYP2130	NcoI_SalI_Hisx6_NcoI_F	F	PCATGGCAGCATCACCATCACCATCACCATCACGGTACCATGGGCGAGCAGAGCTG	ROP10
SYP2131	NcoI_SalI_Hisx6_NcoI_R	R	TCATGGTGATGGTGATGGTGATGGTGATGGTGATGGTGATGGTGATGGTGATGGTGA	ROP10
SYP2132	SpeI_XhoI_SpeI_F	F	PCTAGTCTCGAGTCACTATG	ROP10
SYP2133	attB1_ROP10_CDS_F1	F	GGGGACAAGTTTGTACAAAAAAGCAGGCTTAGGGAAGGCTCCGAAACCATG	ROP10
SYP2134	attB1_ROP10_CDS_F2	F	GGGGACAAGTTTGTACAAAAAAGCAGGCTTTCCGGAAGGCTGTGAGAAGGAACC	ROP10
SYP2135	attB2_ROP10_CDS_R1	R	GGGGACAACTTTGTACAAGAAAGCTGGGTCGCTGAATTCTTCTGAGAGAGATGG	ROP10
SYP2136	attB2_ROP10_CDS_R2	F	GGGGACAACTTTGTACAAGAAAGCTGGGTCGCATTTGTTCGAGGATTTGGATTT	ROP10
SYP2137	ROP10_545F	R	TTTGAAGCTATCTACTTCATC	ROP10
SYP2138	ROP10_60R	F	CAAAATCTCTGCATCATTTTT	ROP10
SYP2139	9xMYCTag_F	R	TTCTGAGGAGGAGGAGGAGGAGGAGGAGGAGGAGGAGGAGGAGGAGGAGGAG	ROP10
SYP2140	ROP9_Knockout_T1_F	F	TTTAAGAAGCTTGCATGAGGAGGAGGAGGAGGAGGAGGAGGAGGAGGAGGA	ROP10
SYP2161	ROP10 F	F	CTTGAGCGTCCATGGAAGTCC	ROP10
SYP2162	ROP10 R	R	TCAATTCTTCCCCAACAGAATG	ROP10
SYP2163	F ROP10 23,27mS	F	CGCGTTGGAAAATTCTCTATCTCTCTCACTCACACG	ROP10
SYP2164	F ROP10 160mS	F	CGGGAGGCTTATTCGAGCTTGTCCTTAAAACCAAG	ROP10
SYP2165	R ROP10 160mS	R	GTTGCTGGTGCTGATCGTGGAAAGTTTTTTTCCAGGC	ROP10
SYP2166	F ROP 1023mS	F	CCGTGAGGGTGGAGATGATGTTTTTCACAG	ROP10
SYP2167	R ROP 1023mS	F	CCGTGAGGGTGGAGATGATGTTTTTCACAG	ROP10
SYP2168	F ROP1070mS	R	CGTTGAAAACCTTGTCCCAAGCTTCCACACG	ROP10
SYP2169	R ROP1070mS	F	CGTTGAAAACCTTGTCCCAAGCTTCCACACG	ROP10
SYP2170	F ROP1027mS	R	CGTTGAAAACCTTGTCCCAAGCTTCCACACG	ROP10
SYP2171	ROP10 cDNA F	F	CTTCTGGGAGGAGGAGGTGTTTCTG	ROP10
SYP2172	ROP10 cDNA R	R	GATCCCAAGCAGATCGAGCTC	ROP10
SYP2173	ROP11 cDNA F	F	CTTGCAATAGTAGGAAGGAGAGGC	ROP10
SYP2174	ROP11 cDNA R	F	TCTAGAGGCGCGCCGGGACTCTT	ROP10
SYP2175	SalI 35SF	R	GAGCTCCAACTCTGTTGAGGTC	ROP10
SYP2176	HindIII NOS Ter R	F	CAAGAGTTGGGAGGTTTTC	ROP10

SYP2179	ROP10 230F	R	CCTGGCTCTGGAGGCTTC	ROP11
SYP2180	ROP10 230R	F	CAACAGTCTGGGGAACCTT	ROP11
SYP2181	F ROP1027mS	R	GAGGGTGAGAGTGGGAGTG	ROP10
SYP2182	R ROP1027mS	F	GTGCTGGGCTAGGGAAG	ROP10
SYP2183	F ROP1070mS	R	GAGGCTCCTCCAGCTG	ROP10
SYP2184	R ROP1070mS	F	GTGTGTGAGTCTGGAGAG	ROP10

Table S4: List of transgenic plant lines created in this study

Name	Genotype	Line	Resistanc	Backgrounc
AtSY2100	*35s:: His$_6$-GFP: AtROP10*	1	Kan	*Col-0*
AtSY2101	*35s:: His$_6$-GFP: AtROP10*	4	Kan	*Col-0*
AtSY2102	*35s:: His$_6$-GFP: Atrop10CA*	1	Kan	*Col-0*
AtSY2103	*35s:: His$_6$-GFP: Atrop10CA*	2	Kan	*Col-0*
AtSY2104	*35s:: His$_6$-GFP: Atrop10$^{C199S\ C205S}$*	2	Kan	*Col-0*
AtSY2105	*35s:: His$_6$-GFP: Atrop10$^{C199S\ C205S}$*	3	Kan	*Col-0*
AtSY2106	*35s:: His$_6$-GFP: Atrop10$^{C199S\ C205S}$*	4	Kan	*Col-0*
AtSY2126	*35s:: His$_6$-GFP: Atrop10CA^{C160S}*	2	Kan	*Col-0*
AtSY2127	*35s:: His$_6$-GFP: Atrop10CA^{C160S}*	4	Kan	*Col-0*
AtSY2128	*35s:: His$_6$-GFP: Atrop10$^{\Delta 183\text{-}197}$*	1	Kan	*Col-0*
AtSY2129	*35s:: His$_6$-GFP: Atrop10$^{\Delta 183\text{-}197}$*	2	Kan	*Col-0*
AtSY2130	*35s:: His$_6$-GFP: Atrop10$^{\Delta 200\text{-}204}$*	1	Kan	*Col-0*
AtSY2131	*35s:: His$_6$-GFP: Atrop10$^{\Delta 200\text{-}204}$*	3	Kan	*Col-0*
AtSY2144	*35s:: His$_6$-GFP: Atrop10CA^{C23S}*	1	Kan	*Col-0*
AtSY2145	*35s:: His$_6$-GFP: Atrop10CA^{C23S}*	2	Kan	*Col-0*
AtSY2146	*35s:: His$_6$-GFP: Atrop10^{C160S}*	1	BAR	*Col-0*
AtSY2147	*35s:: His$_6$-GFP: Atrop10^{C160S}*	2	BAR	*Col-0*

Supplementary Figures

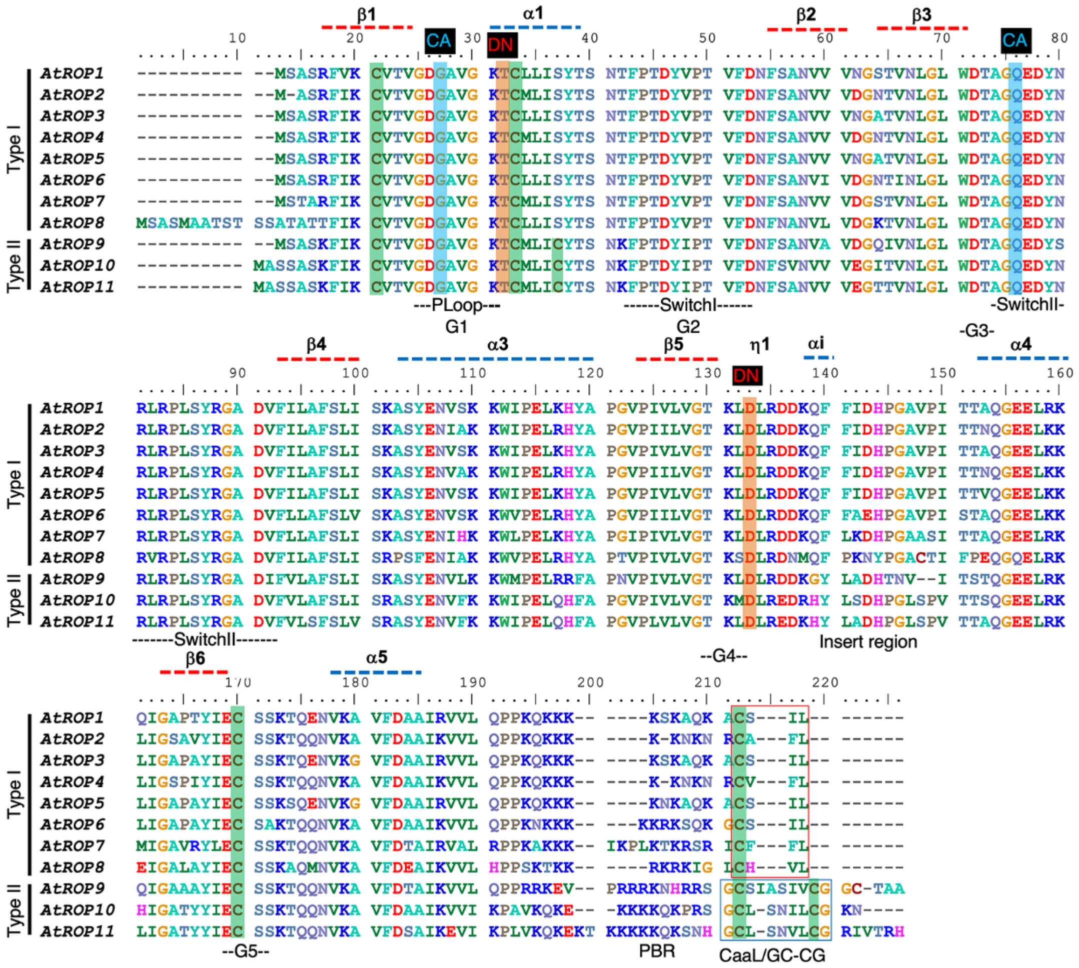

Fig S1. Multiple Sequence Alignment between ROP1-10. Multiple Sequence Alignment (MSA) was performed using MAFFT (http://mafft.cbrc.jp/alignment/server). The resultant alignment was subsequently processed with BioEdit 7.1.3. The positions of cysteines C11, C23, C27, C160, C199, and C205 are annotated and highlighted in green (these positions correspond to ROP10). The positions of constitutively active (CA) and dominant negative (DN) mutants are also marked and highlighted in blue and orange, respectively. The CaaX box and GC-CG box motifs are represented by red and blue rectangles, respectively. The G1-G5, α-helices, β-sheet, PLoop, SwitchI/II, Insert region, and the poly basic region (PBR) are annotated in their respective positions. The alignment is specific to *Arabidopsis*.

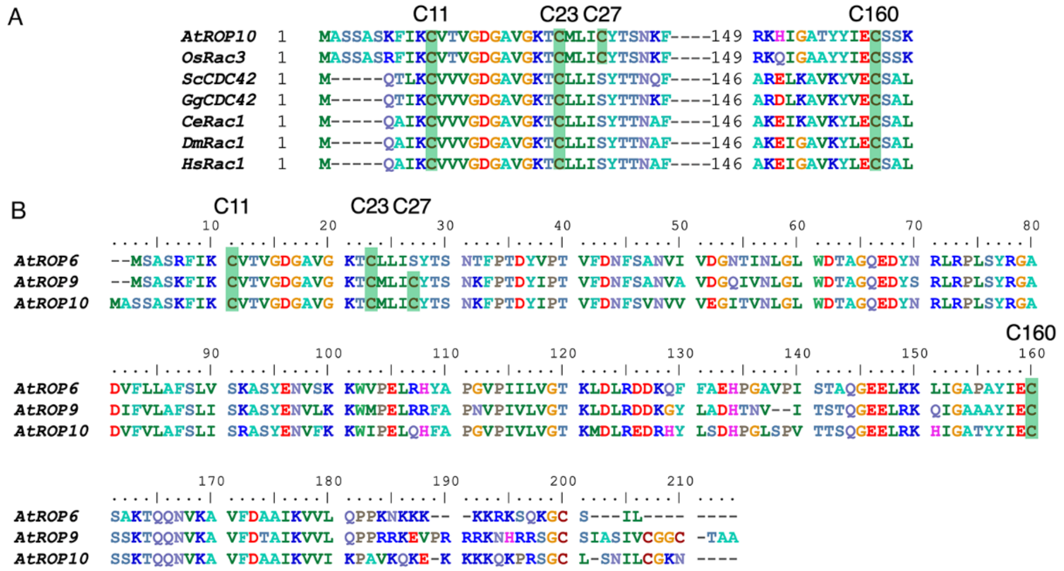

Fig S2. Multiple Sequence Alignment. A) Multiple Sequence Alignment (MSA) a of the G-domains of ROP10 and non-plant Rho family GTPases. The MSA was conducted using MAFFT (http://mafft.cbrc.jp/alignment/server). The results were processed with BioEdit 7.1.3. The positions of cysteines C11, C23, C27, and C160 are marked and highlighted in green. **B)** MSA of *Arabidopsis* ROP6, ROP9, and ROP10. The MSA was performed using MAFFT (http://mafft.cbrc.jp/alignment/server). The results were processed with BioEdit 7.1.3. The positions of cysteines C11, C23, C27, and C160 are marked and highlighted in green.

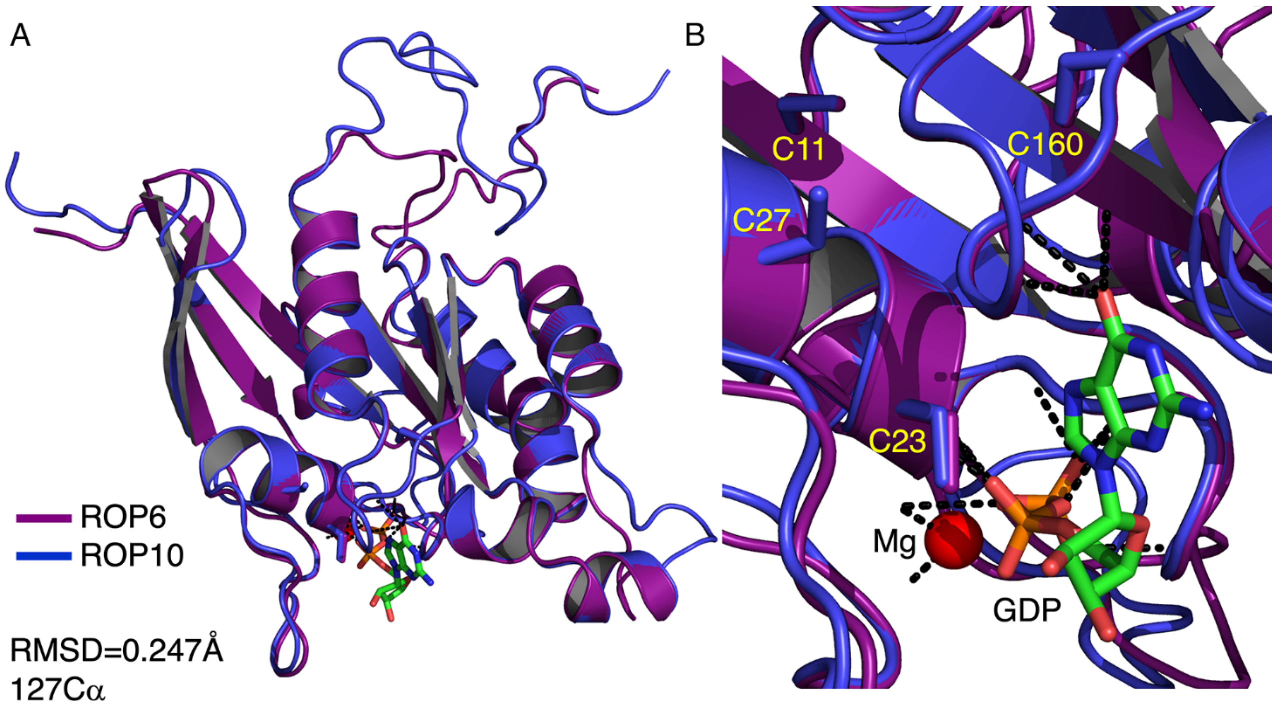

Fig S3. Superposition of ROP6 and ROP10. Superposition and root mean square deviation (RMSD) calculation of ROP6 and ROP10 were performed using PyMol (http://pymol.sourceforge.net), employing default parameters. The calculated RMSD value was 0.247 Å, encompassing 127 Cα. **A)** An overview of the superposition between the two proteins, with purple representing ROP6 and blue representing ROP10. **B)** Zoom-in on the nucleotide binding pocket, highlighting C11, C23, C27, C160, Mg, and GDP. The dotted black line indicates the interaction between GDP and a specific amino acid within the protein.

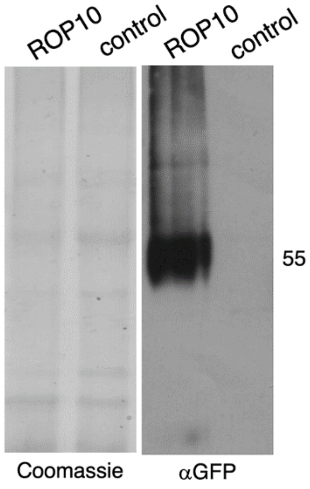

Fig S4. Purification of His$_6$-GFP-ROP6 from Transgenic *Arabidopsis*. Left: Coomassie blue-stained gel of ion-exchange-enriched fractions purified from His$_6$-GFP-ROP10 expressing plants (ROP10) and control non-transgenic plants. Right: Protein immunoblot decorated with anti-GFP antibodies. The number on the right denotes the molecular weight in kilodaltons.

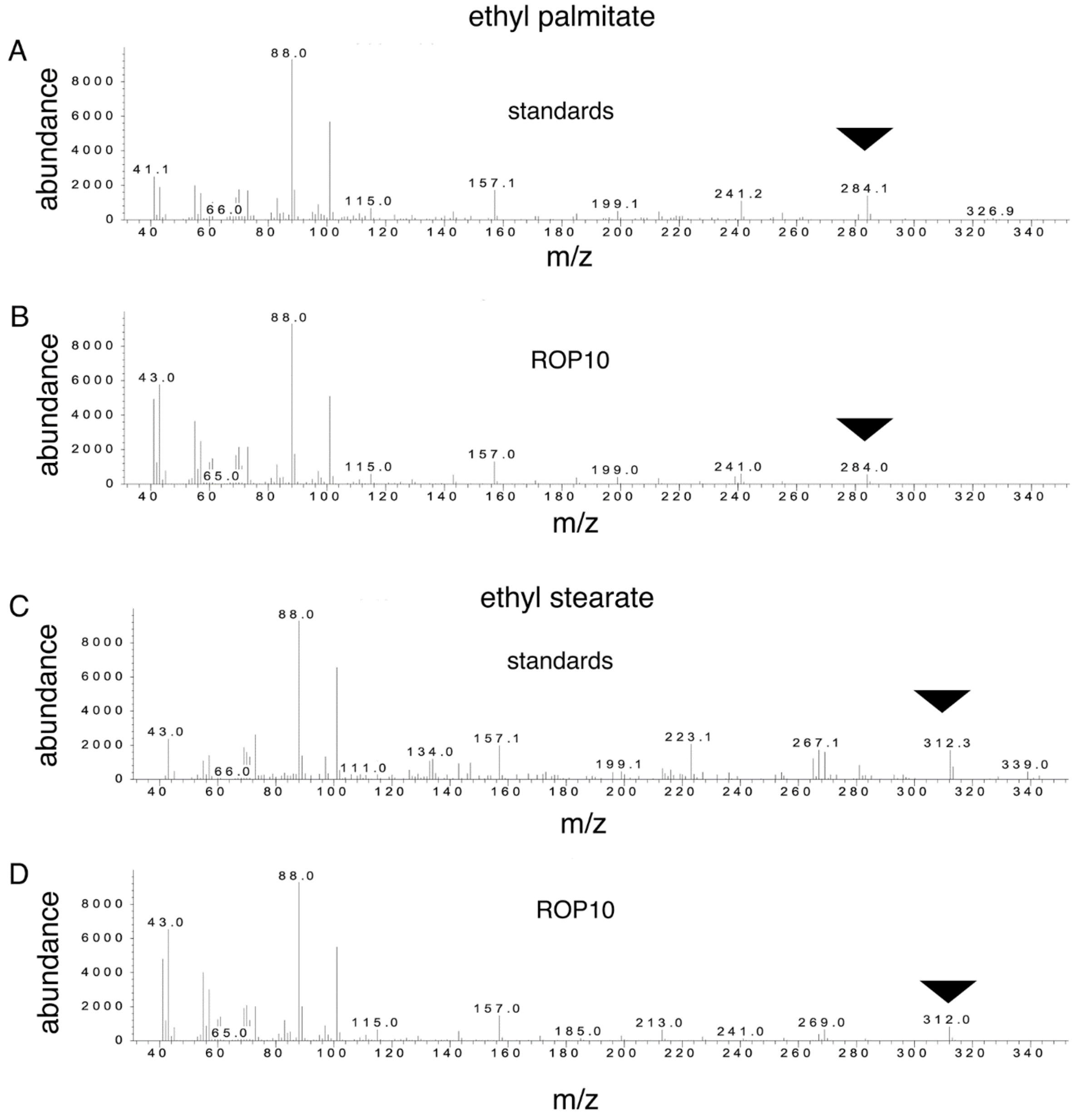

Fig S5. MS chromatograms of the GC identified fatty acids. A) Ethyl palmitate standards, **B)** ethyl palmitate purified from ROP10. **C)** ethyl stearate standards. **D)** Ethyl stearates purified from ROP10. Arrowheads indicate m/z values of the typical molecular ions: ethyl palmitate- 284 and ethyl stearate- 312. m – mass, z – charge.

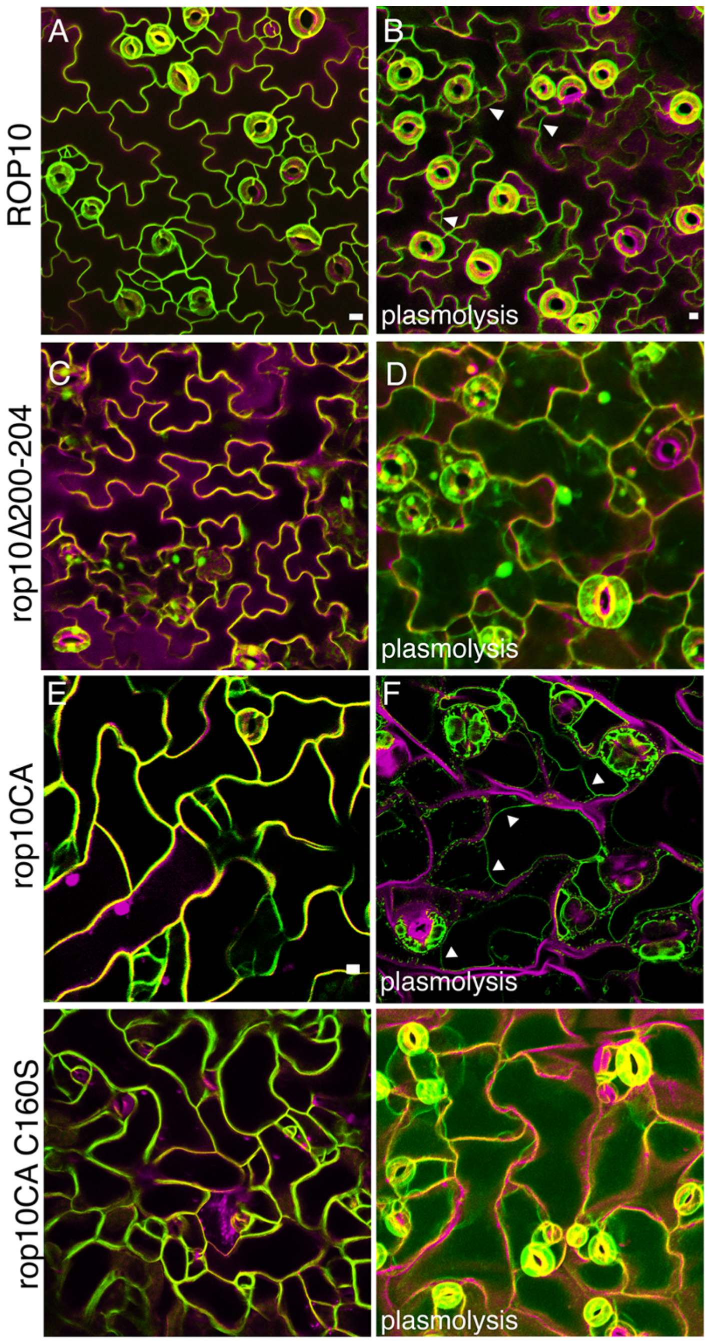

Fig. S6. Subcellular localization of ROP10 and rop10 mutant proteins in stably expressing *Arabidopsis* plants. Enlarged images corresponding to Figures 3 and 4 in the main text are shown. Green indicates GFP fluorescence; magenta indicates propidium iodide (PI) staining. Arrowheads in panels B and F indicate plasma membrane regions detached from the cell wall.

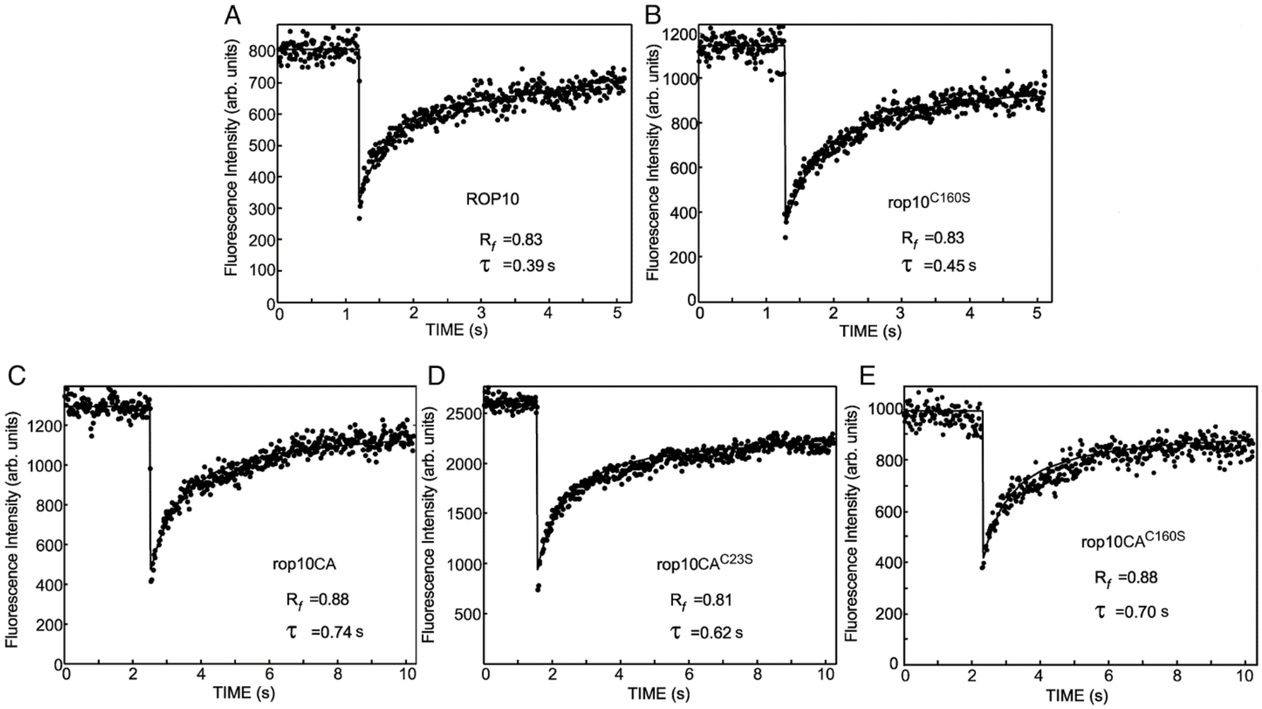

Fig S7. Representative FRAP curves of GFP-ROP10 variants. The curves shown were all obtained with the 63x objective, to enable direct comparison between the mutants. All the experimental data points (fluorescence intensity vs. time) are shown within each curve; the high initial counts at the left portion of each curve show the pre-bleach level measured with the low-intensity monitoring beam, the sharp decrease in fluorescence depicts the short bleach with a high-intensity laser beam, and the gradual increase in the intensity upon shifting back to the monitoring beam shows the recovery phase. **A)** ROP10. **B)** rop10^{C160S}. **C)** rop10CA. **D)** rop10CA^{C23S}. **E)** rop10CA^{C160S}. The solid lines show the best fit (nonlinear regression analysis) to the lateral diffusion equation. The R_f (mobile fraction) and τ values derived for each specific curve are shown within each panel. Note that the scales of the time axis differ between panels A, B and panels C-E, as the latter yield slower fluorescence recovery.

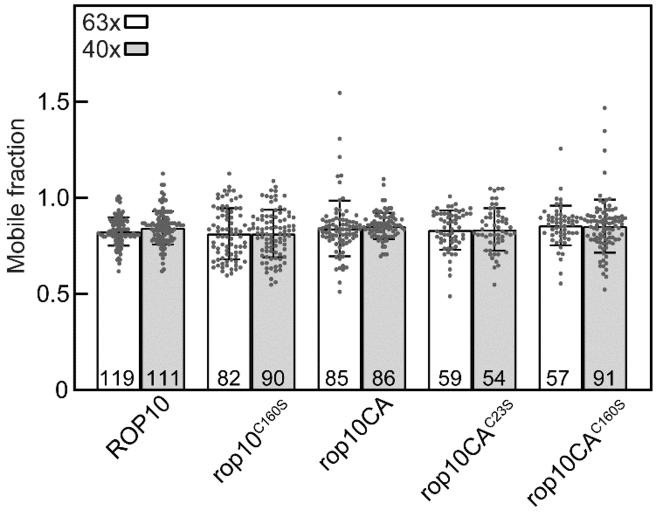

Fig S8. FRAP beam size analysis shows similar mobile fractions for all ROP10 variants measured. For each ROP10 variant, the specific transgenic lines employed are identical to those designated in the legend to Fig 7. Experiments were conducted as described in Fig 6. Bars are means ± SD of multiple independent measurements, each conducted on a different cell (the number of measurements is depicted within each bar). No significant differences were found between the mobile fraction values of all ROP10 variants (one way ANOVA and Tukey's post-hoc test).

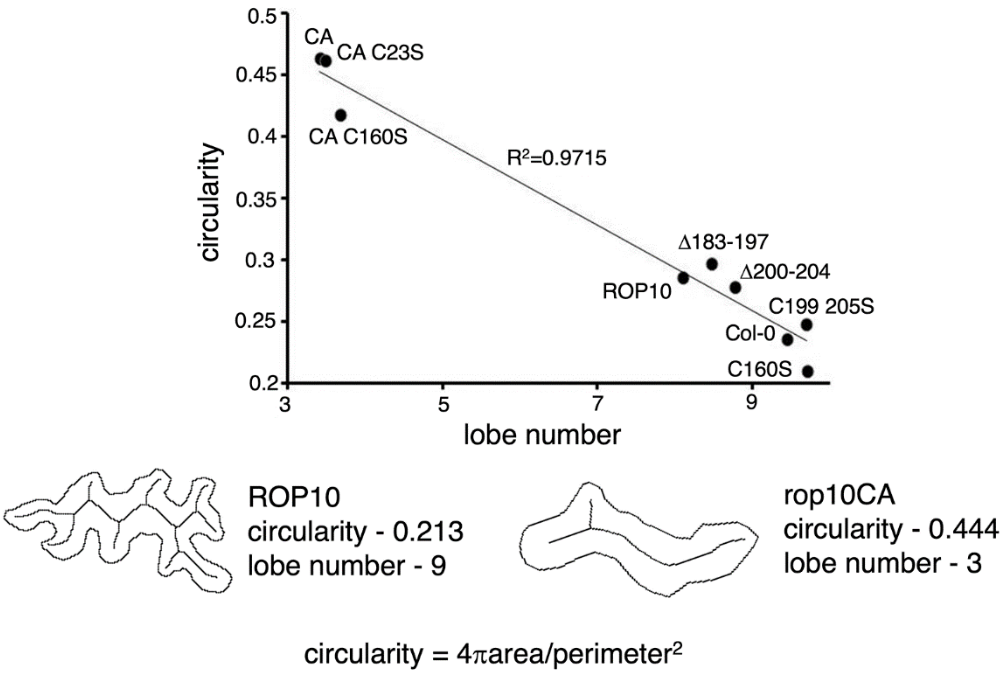

Fig S9. Inverse linear correlation between circularity and lobe number in ROP10 wild-type mutants. Top: A plot demonstrating the nearly inverse linear relationship between circularity and lobe number (R^2=0.9715). *Col-0*, ROP10, rop10CA (CA), rop10CA^{C23S} (CA C23S), rop10^{C160S} (C160C), rop10CA ^{C160S} (CA C160S), rop10$^{\Delta 183\text{-}197}$ (Δ183-197), rop10$^{\Delta 200\text{-}204}$ (Δ200-204), rop10$^{C199S\ C205S}$ (C199 205S). Bottom: Representative cell diagrams employed for circularity and lobe count calculations. The thin black line within each cell signifies the skeletonization utilized for determining lobe count.

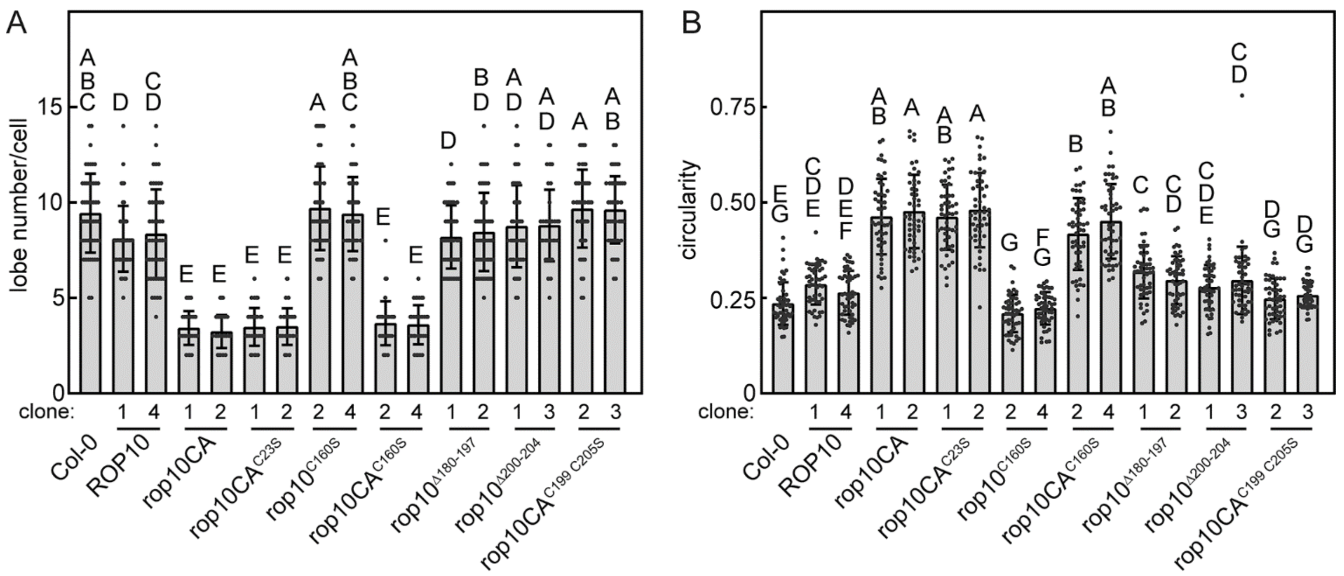

Fig S10. The intact hypervariable domain and G domain cysteine C160 are crucial for the influence of ROP10 on cellular structure. A) lobe number; **B)** circularity. Error bars - SD. Letters above bars in all correspond to statistically significant difference (one-way ANOVA and Tukey's HSD test, $p \leq 0.05$). Representative images are shown in Figs. 3 and 4. Fifty cells were measured for each cell line. For each ROP10 variant, two independent transgenic lines were analyzed. This figure is related to Fig 7, which presents data for a single line (the first line from the left for each variant) from each genotype.

References

1. Earley KW, Haag JR, Pontes O, Opper K, Juehne T, Song K, et al. Gateway-compatible vectors for plant functional genomics and proteomics. Plant J. 2006;45(4):616-29. Epub 2006/01/31. doi: TPJ2617 [pii]

10.1111/j.1365-313X.2005.02617.x. PubMed PMID: 16441352.

2. Lavy M, Bracha-Drori K, Sternberg H, Yalovsky S. A Cell-Specific, Prenylation-Independent Mechanism Regulates Targeting of Type II RACs. Plant Cell. 2002;14(10):2431-50. PubMed PMID: 12368496.
