# Supplementary material for: C-terminal S-acylation governs membrane distribution, interaction dynamics and function of a plant Rho GTPase
Source: PLoS One. 2026 Apr 30;21(4):e0348444. doi: 10.1371/journal.pone.0348444 (PMC13132179; doi:10.1371/journal.pone.0348444)
Supplement: S2 File — (PDF) [file pone.0348444.s002.pdf]

Raw Images of immune blots from figure 5

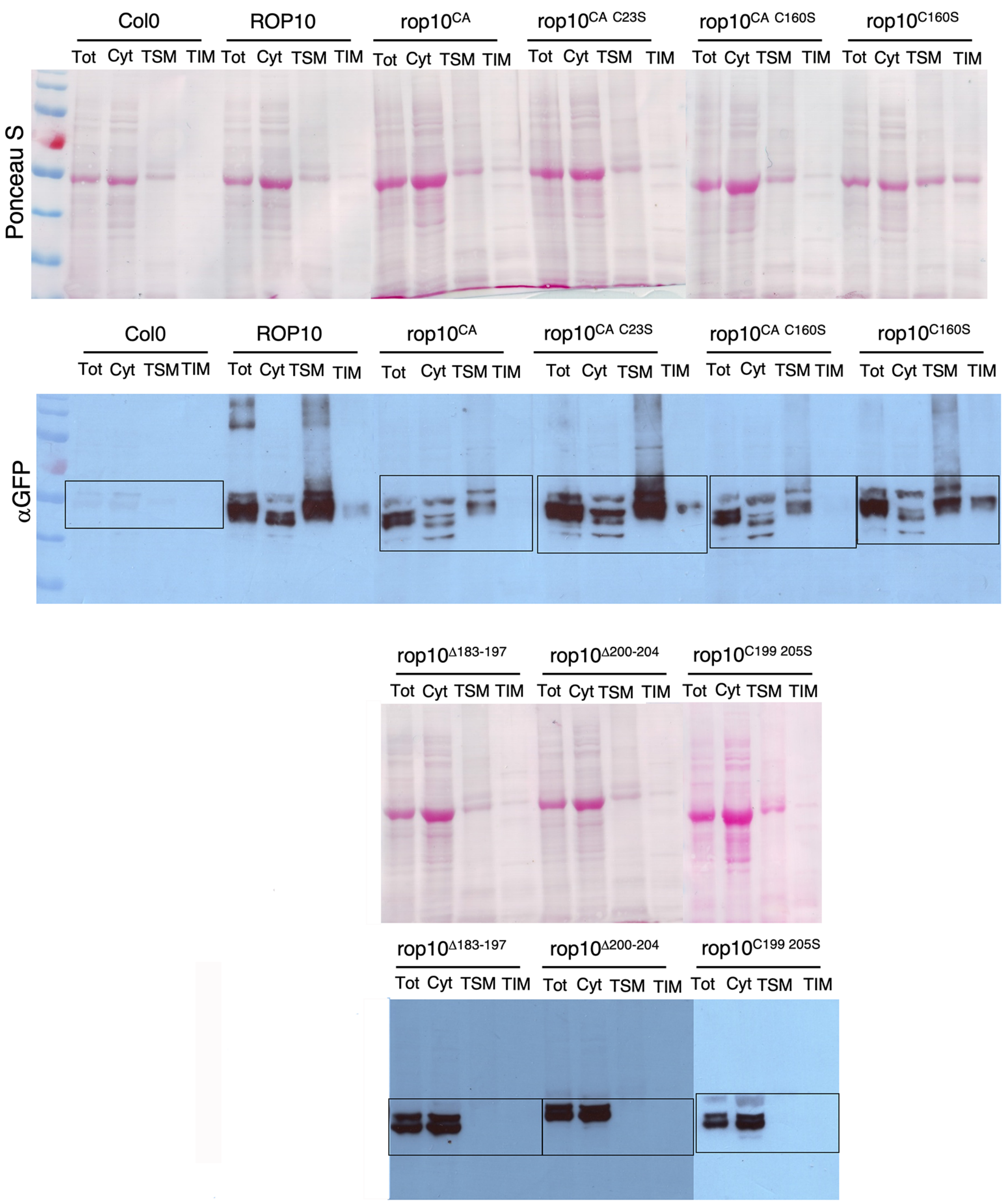

Fig. S1. Full immunoblots corresponding to Fig. 5 (main text). The boxed regions indicate the areas shown in the figure.
